# Effect of Grinding and the Mill Type on Magnetic Properties of Carboxylated Multiwall Carbon Nanotubes

**DOI:** 10.3390/ma14144057

**Published:** 2021-07-20

**Authors:** Agnieszka Jamrozik, Janusz Przewoznik, Sonia Krysiak, Jozef Korecki, Grzegorz Trykowski, Artur Małolepszy, Leszek Stobiński, Kvetoslava Burda

**Affiliations:** 1 Institute of Physical Chemistry, Polish Academy of Sciences, ul. Kasprzaka 44/52, 01-224 Warsaw, Poland; ajamrozik@ichf.edu.pl; 2 Faculty of Physics and Applied Computer Science, AGH—University of Science and Technology, al. Mickiewicza 30, 30-059 Kraków, Poland; januszp@agh.edu.pl (J.P.); Sonia.Krysiak@fis.agh.edu.pl (S.K.); 3 Jerzy Haber Institute of Catalysis and Surface Chemistry, Polish Academy of Sciences, ul. Niezapominajek 8, 30-239 Krakow, Poland; korecki@agh.edu.pl; 4 Faculty of Chemistry, Nicolaus Copernicus University in Torun, ul. Gagarina 7, 87-100 Torun, Poland; tryki@chem.umk.pl; 5 Faculty of Chemical and Process Engineering, Warsaw University of Technology, ul. Waryńskiego 1, 00-645 Warsaw, Poland; Artur.Malolepszy@pw.edu.pl (A.M.); leszek.stobinski@pw.edu.pl (L.S.)

**Keywords:** multiwall carbon nanotubes, functionalization, nanoparticles, iron phases, milling, magnetic properties, effective anisotropy constant

## Abstract

The influence of the grinding process on the magnetic properties of *as prepared* and functionalized multiwall carbon nanotubes (MWCNTs) is presented. We have observed that 3 h mechanical grinding at 400 rpm in contrast to functionalization does not remove the iron contamination from MWCNTs. However, it changes the Fe chemical states. The magnetic properties of iron nanoparticles (Fe-NPs) embedded in the carbon matrix of MWCNTs have been analyzed in detail. We have proven that single-domain non-interacting Fe_(C,O)_-NPs enriched in the Fe_3_C phase (~10 nm) enclosed inside these nanotubes are responsible for their magnetic properties. Mechanical grinding revealed a unique impact of -COOH groups (compared to -COONH_4_ groups) on the magnetism of functionalized MWCNTs. In MWCNT-COOH ground in a steel mill, the contribution of the Fe_2_O_3_ and α-Fe phases increased while the content of the magnetically harder Fe_3_C phase decreased. This resulted in a 2-fold coercivity (*H_c_*) decrease and saturation magnetization (*M_S_*) increase. A 2-fold remanence (*M_r_*) decrease in MWCNT-COOH ground in an agate mill is related to the modified Fe_(C,O)_-NP magnetization dynamics. Comparison of the magnetostatic exchange and effective anisotropy length estimated for Fe_(C,O)_-NPs allows concluding that the anisotropy energy barrier is higher than the magnetostatic energy barrier. The enhanced contribution of surface anisotropy to the effective anisotropy constant and the unique effect of the -COOH groups on the magnetic properties of MWCNTs are discussed. The procedure for grinding carboxylated MWCNTs with embedded iron nanoparticles using a steel mill has a potential application for producing Fe-C nanocomposites with desired magnetic properties.

## 1. Introduction

Carbon nanotubes (CNTs) were first observed in 1991 [1]. Nobody expected that these nanocylindrical forms of pure carbon will reveal so many uncommon properties and will be the important object of interest in modern technologies [2], for example, as sensors or drug containers in nanomedicine [3,4], catalysts and energy converters [5,6], or high-performance microwave absorption materials (MAMs) for environmental, medical, and military applications [7]. Hydrophobicity and chemical inertness often constitute an obstacle to a wide range of CNTs applications when their solubility in polar solvents is required. Non-functionalized CNTs can easily aggregate to form agglomerates stabilized by van der Waals interactions [8]. Iron contaminants can further hold together CNT stacks due to magnetic interactions [9]. Chemical functionalization allows modification of the surface and core of CNTs, increasing the compatibility of nanotubes with other materials and modulation of their electronic [10,11] and mechanical properties [12]. The oxidation process of CNTs has gained a lot of attention, particularly in an attempt of the purification of these structures. Deagglomeration of CNTs bundles due to the presence of oxygen groups increased their hydrophilicity. This also facilitates further CNT modification required for specific applications [13,14]. The functionalization of multiwall carbon nanotubes (MWCNTs) has been less studied compared to singlewall carbon nanotubes (SWCNTs) because they are less reactive and more difficult to disperse. Therefore, knowledge of the impact of functionalization methods on different properties of MWCTs is crucial for the further development of their potential applications [11,15]. In particular, their electronic and magnetic properties could be exploited in modern nanotechnologies [2,16]. Morphology and the high aspect ratio of CNTs have made carbon nanotubes attractive nanocontainers for magnetically ordered phases [17,18]. Loading magnetic nanoparticles (NPs) into the carbon nanotube channel ensures a narrow distribution of their size and prevents inter-particle interactions. It is expected that the reduced transverse CNT dimension can increase the NP elongation, magnetic shape anisotropy, coercive field, and then stabilize the magnetic order against thermal fluctuations. The filling of CNTs with magnetic elements, such as iron or other 3d-metals, makes these systems potential candidates for application in nanodevices (for example, for high-resolution magnetic field sensors) and high-density magnetic memory materials [19]. Therefore, interest in wire type structures has increased. Several methods have been described that can be used to introduce metals into carbon nanotubes, and some of them simply rely on the dc arc evaporation commonly used for the synthesis of carbon nanotubes in the presence of a ferromagnetic metal, for example, of iron [20]. Usually, the α-Fe, γ-Fe, and Fe_3_C (cementite) phases were observed in the iron-filled CNTs, but their relative content in the formed iron nanostructures depends mainly on the procedure used. It has been revealed that α-Fe as the initial active phase and Fe_3_C as the main carbide play a special role in creating catalytically prepared CNTs [21]. Moreover, it was demonstrated that the properties of magnetic nanomaterials could be altered beyond the use of chemical functionalization by applying mechanical milling or blocking small nanoparticles in rigid networks [22,23].

Iron carbides are attractive magnetic soft materials for the production of composites characterized by large energy products ((BH)_max_) [24]. Therefore, a great deal of effort is put into developing procedures of filling carbon nanotubes with metals to improve their magnetic [22] and catalytic properties [25]. Mechanical milling was successfully applied to improve the magnetic properties of many magnetic materials [26,27]. The increase in strain and density of structural defects in milled samples containing mixed hard and soft materials leads to the emergence of a new nanomaterial with the high saturation magnetization of the soft phase and the high coercivity of the hard phase. It was also found that the CNT inclusions in composite materials caused by high-energy milling leads to an improvement in their thermal, electrical, and mechanical properties [28,29,30].

In this work, we present studies of the magnetic properties of the iron inclusions in non-functionalized and functionalized MWCNTs (containing -COOH and -COONH_4_ groups), ground in an agate or steel mill cylinder used at the end of the preparation procedure. Iron was built into these MWCNTs during their synthesis because it was used as a catalyst. We observed that the functionalization and milling treatments cannot completely remove iron impurities but can modify the iron states inside MWCNTs and hence their magnetic properties. In particular, we found a large impact of grinding on MWCNT -COOH magnetism. We were also looking for changes in Fe-NPs and their interactions with the interior of the carbon nanotubes. We present a detailed analysis of chemical and magnetic properties of the Fe-NPs responsible for the magnetism of MWCNTs. Mössbauer spectroscopy was used to monitor valence and spin states of iron atoms and a local magnetic field of iron compounds. Vibrating sample magnetometry (VSM) gives information on the global magnetic properties of MWCNTs.

We present here a simple combination of the chemical and mechanical treatments for Fe-MWCNTs that can directly improve Fe-NP magnetic properties. The presented results indicate an easy and efficient way for magnetic nanocomposites production, which can find a wide application in future nanotechnology.

## 2. Materials and Methods

### 2.1. Sample Preparation

**Figure 1:**
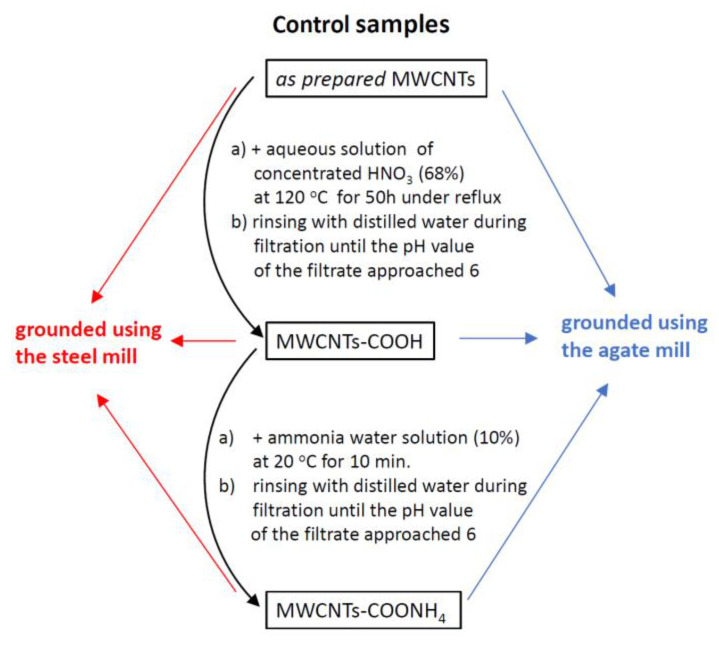
Steps of MWCNTs functionalization and their grinding.

We investigated non-functionalized MWCNTs (Ctube100, CNT Co., Ltd., South Korea) called *as prepared* MWCNTs and their two functionalized forms: carboxylated MWCNTs—COOH and ammonium salt of carboxylated nanotubes MWCNTs—COONH_4_. Steps of their functionalization are presented in Figure 1.

In the final preparation step, these three types of MWCNTs were ground mechanically using a planetary ball mill (Retsch PM 100, Haan, Germany). A steel vessel and steel balls or an agate vessel and agate balls were used. The mass ratio of CNTs to balls was 1:10. Each run lasted 3 h under the centrifugation of 400 rpm. Properties of the grinding jars and balls are shown in Table 1.

In this way, we obtained the following configurations of samples: (i) control group (*as prepared* MWCNTs, MWCNTs—COOH and MWCNTs—COONH_4_, (ii) *as prepared* MWCNTs, MWCNTs—COOH and MWCNTs—COONH_4_ triturated using an agate mill and (iii) *as prepared* MWCNTs, MWCNTs—COOH and MWCNTs—COONH_4_ triturated using a steel mill.

### 2.2. Methods

All of the types of nanotubes presented in Figure 1 were investigated using:

(i)  Mössbauer spectroscopy (spectra were recorded at a home-made cryostat (Kraków, Poland) at 85 K, 220 K and 295 K; ΔT = 0.1 K; a source of γ—radiation with an energy of 14.4 keV: ^57^Co(Rh); an absorption spectrum of α-Fe at room temperature was used for the calibration).

Information about the spin and valence states of iron, the type of Fe ligands, and their organization, and magnetic properties of the iron compounds. Hyperfine parameters were obtained using Recoil software [31].

(ii)  the Vibrating Sample Magnetometer (VSM) option of a 9 T Quantum Design Physical Property Measurement System (PPMS), (Quantum Design North America, San Diego, California); (temperature measurements within a wide range from 3 K to 350 K; the external field (μ_0_*H*) used: up to ± 8 T).

Investigations of the dc magnetic moment (μ) and mass magnetization (*M*). Samples with typical mass 10–20 mg were placed in two parts, head-to-tail (magnetically clean) polypropylene powder cells installed on the brass half-tube VSM sample holder.

(iii)  a high-resolution transmission electron microscope (TEM), G2 F20X-Twin 200 kV, (FEI, Brno, Czech Republic) equipped with a Si(Li) detector SUTW, 136 eV (EDAX, Mahwah, USA) for recording of energy dispersive X-ray spectra (EDX).

Characterization of the size and structure of CNTs, metal inclusions, and analysis of the chemical composition of the particles.

(iv) Inductively Coupled Plasma—Optical Emission Spectrometer (ICP-OES), *Optima 7000 DV ICP-EOS* (PerkinElmer, Waltham, MA, USA).

Measurements of the Fe and other metals content in MWCNTs using the microwave digestion method (MD, HNO_3_-H_2_O_2_) [32].

## 3. Results

### 3.1. Mössbauer Experiments

**Figure 2:**
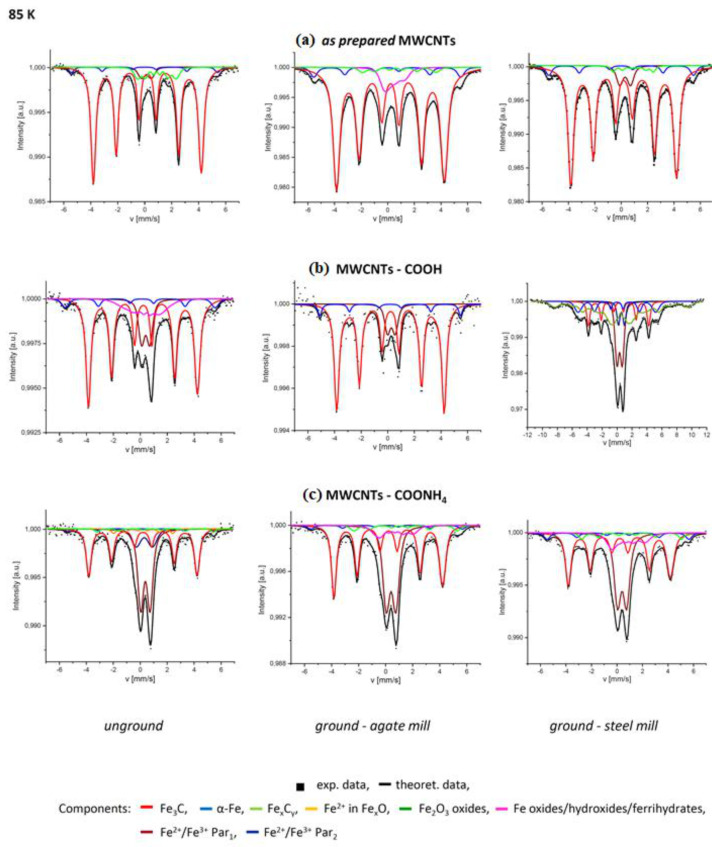
Mössbauer spectra for (**a**) *as prepared* MWCNTs, (**b**) MWCNTs-COOH, and (**c**) MWCNTs-COONH_4_: left column —the control group, middle column—after using the agate mill, right column—after using the steel mill, all measured at 85 K. In the case of MWCNTs-COOH treated by a steel mill (b—right column), we applied a wider velocity scale due to the presence of the magnetically ordered iron oxide phase with a high hyperfine magnetic field.

The Mössbauer spectra for the control group of unground nanotubes (*as prepared* MWCNTs, MWCNTs-COOH, MWCNTs-COONH_4_) and MWCNTs ground by use of the agate and steel mill measured at 85 K are shown in Figure 2. Corresponding hyperfine parameters fitted to these experimental data collected in Table S1 and the experimental and theoretical data obtained for the samples at 220 K and 295 K are included in Supplementary materials (Figures S1 and S2; Tables S2 and S3).

The Mössbauer data show that not only functionalization but also milling caused significant changes of the state of iron compounds embedded inside MWCNTs.

#### 3.1.1. The Control Group of MWCNTs

In order to obtain good fits of the Mössbauer spectra of the control *as prepared* and carboxylated MWCNTs, at least four components had to be taken into account. In the case of ammonium nanotubes, five components were necessary (Figure 2 and Figures S1 and S2, left columns); a dominating magnetically splitted subspectrum, with an isomer shift of about 0.2 mm/s, a magnetic hyperfine field *H_hf_* ≈ 25 T, and a very small quadrupole splitting has hyperfine parameters characteristic of cementite, Fe_3_C. Part of cementite (20%) had a large distribution of Δ*H_hf_* ≈ 9.6 T, indicating structural disorders in the Fe_3_C clusters. Cementite had the largest contribution in all spectra of non-functionalized nanotubes (about 82% at 85 K). The MWCNTs-COOH content dominated at the level of 66% but in MWCNTs-COONH_4,_ it decreased to ~42% (at 85 K).

The magnetically splitted subspectra with an isomer shift close to 1 mm/s at 85 K present in *as prepared* and ammonium nanotubes were unambiguously related to the high spin Fe^2+^ states. Among naturally occurring iron compounds, which could be consistent with the composition of the present samples, similar hyperfine parameters were characteristic of wüstite (non-stoichiometric iron oxide Fe_1−x_O) with a complex defect and magnetic structure—an antiferromagnet with T_N_ of approximately 200 K [33]. The properties of wüstite-like phases became even more complicated at the nanoscale [34], and, additionally, if such an oxide layer formed on a ferromagnet, its proximity can significantly enhance the Néel temperature in FeO [35]. The above picture can explain the whole range of spectral components, labelled as Fe_x_O in Table 1, with variable magnetic splitting (from 8.9 ± 1.1 T detected for *as prepared* CNTs up to 26.8 ± 0.1 T for MWCNTs-COONH_4_) and the isomer shift around 1 mm/s. This Fe-phase was not observed in carboxylated nanotubes but another one with magnetic ordering appeared (*H_hf_* ≈ 9 T) and IS ≈ 0.4 mm/s (at 85 K). It originated from small iron oxides/oxyhydroxides or ferrihydrates clusters [36]. In the case of MWCNTs-COONH_4_, a subspectrum with *IS* ≈ 0.19 mm/s, a small quadrupole splitting and *H_hf_* ≈ 14 T (at 85 K) were found. These hyperfine parameters allowed us to assign it to Fe-C compounds in which the number of C atoms in the Fe vicinity increased compared to cementite [37]. There was also a small α-Fe component (about 4% in *as prepared* CNTs and those functionalized with ammonium groups, about 7% in the caboxylated CNTs) present in the samples. The paramagnetic components (Par) with a broad quadrupole splitting distribution (*QS* ~0.60–1.20 mm/s and Δ*Q* ~0.22–0.50 mm/s) observed in the spectra can be assigned to Fe(C) oxides/oxyhydroxides or ferrihydrates [38,39,40]. The *Par* content in *as prepared*, carboxylated, and ammonium nanotubes was about 5%, 14%, and 50% (at 85 K), respectively.

#### 3.1.2. MWCNTs Ground in the Agate Mill

In this group, all MWCNTs (*as prepared*, MWCNTs-COOH, MWCNTs-COONH_4_) were ground using the agate mill (Figure 2 and Figures S1 and S2, middle columns). In the ground *as prepared* CNTs, the fraction of cementite was at the same level as in the untreated nanotubes, but in functionalized carboxylated and ammonium nanotubes, it increased to about 83% and 49% (at 85 K), respectively. α-Fe was also present in all MWCNTs and its content was almost the same as that in the unground nanotubes. Milling resulted in the disappearance of the paramagnetic oxides/oxyhydroxides/ferrihydrate phase in *as prepared* MWCNTs and occurrence of its magnetically ordered phase with *H_hf_* ≈ 5 T and Δ*H_hf_* ≈ 1.6 T (at 85 K). In ground MWCNTs-COOH such magnetically ordered oxide/oxyhydroxide or ferrihydrate phase disappeared, and the paramagnetic phase contribution in the spectra decreased by almost 4%. In the case of ground MWCNTs-COONH_4_, the paramagnetic component decreased by about 15%. At the same time, a new magnetically ordered phase occurred, characterized by *IS* ≈ 0.5 mm/s and *H_hf_* ≈ 9 T (at 85 K). It can be assigned to the mixed spin and valence states of Fe(C) oxides/oxyhydroxides [36,41]. In the ground *as prepared* and ammonium nanotubes, the ferrous magnetically ordered phase Fe_x_O remained. However, in the first case, its magnetic hyperfine field increased about 2-fold whereas its contribution decreased almost 3-fold.

#### 3.1.3. MWCNTs Ground in the Steel Mill

For the *as prepared* MWCNTs, the same iron phases as in the unground sample were observed after grinding in the steel mill. Only the contribution of the paramagnetic and α-Fe components increased, each by about 2%, while that of the ferrous phase decreased by about 6%. In the case of ground MWCNTs-COONH_4_, the same iron phases were observed as for these nanotubes ground in the agate mill. The low field magnetic component of iron oxides/oxyhydroxides or ferrihydrates was detected but with a slightly higher isomer shift (by about 0.10 mm/s) at 85 K (there was no difference at RT), and its contribution increased to about 12%. The most striking differences in the use of the steel mill instead of the agate one were observed for MWCNTs-COOH (Figure 2 and Figures S1 and S2, right columns). First, the content of the Fe_3_C phase decreased to about 16%, and a magnetically ordered oxide/hydroxide phase occurred, of which the contribution increased to about 42% (at 85 K). The contribution of the latter one decreased with increasing temperature, and at RT was at the level of about 29%. This component was characterized by a wide distribution of the hyperfine magnetic field 10 T < *H_hf_* < 55 T at 85 K and 10 T < *H_hf_* < 30 T at RT. The contribution of the component with the highest field (55 T) at the low temperature was about 8%. Such a high magnetic field is a theoretical limit for an Fe^3+^ ion [41] and can be expected for Fe_2_O_3_ compounds (hematite or maghemite) with magnetic properties enhanced by surface effects in nanomaterials [42]. This fraction disappeared at higher temperatures, which means that it formed by superparamagnetic particles. The superparamagnetic phase contributed to the so-called *Par* phase, of which the content increased at RT by almost 10% (Tables S1–S3). The content of the α-Fe fraction increased to 12% and 19% at 85 K and RT, respectively, in the carboxylated nanotubes triturated in the still mill. Therefore, its content increased about 2-fold in comparison to the MWCNTs unground and ground in an agate mill.

**Figure 3:**
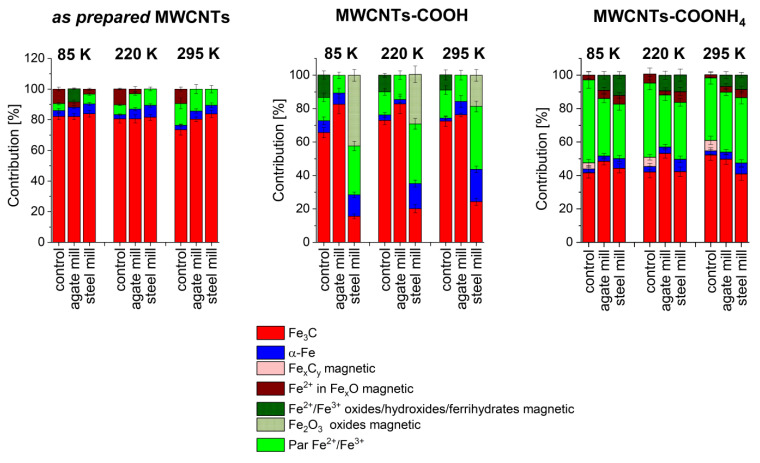
Contributions of iron phases in non-functionalized and functionalized MWCNTs ground in the agate and steel mill.

Temperature changes of subsequent component contributions with increasing temperature for unground and ground MWCNTs are presented in Figure 3. As one may expect, the values of hyperfine magnetic fields obtained for the subsequent magnetic components decreased with increasing temperature in all investigated groups of these carbon nanotubes (Tables S1–S3).

### 3.2. VSM Experiments

The dc magnetization measurements give information on the global magnetic properties of MWCNTs that are related to their carbon matrix and built-in magnetically ordered Fe-complexes. Mass magnetization (*M*) values were corrected by subtracting the diamagnetic response of the carbon nanotubes from the total magnetization of the experimental data using the results from measurements of the high-purity graphite sample. The experiment was carried out in two mutually perpendicular orientation of the rectangular parallelepiped-shaped graphite specimen (and then the average value of the diamagnetic response was calculated) under the same experimental conditions (at the external magnetic field 4 T and the temperature between 4 and 350 K). The measured diamagnetic moment for graphite, scaled to the carbon content in a particular sample, was subtracted from the total moment of the experimental data points.

To correctly calculate mass magnetization, the mass of the sample was corrected for the carbon contribution knowing the carbon and Fe content from ICP-OES measurements (Table S4 in Supplementary Materials). In Supplementary Materials in Figure S3, the magnetic moment (μ) measured for these nanotubes in the field of 4 T as a function of temperature is shown. One can note that the contribution of carbon to μ values was quite similar and important for the two functionalized MWCNTs-COOH samples. The μ vs. *T* dependences revealed generally similar behaviors at higher temperature as expected for ferromagnetic materials but at low temperatures, a much stronger increase of μ was observed for the sample prepared in the steel mill, indicating a much larger contribution of the superparamagnetic phase in this case.

The thermal evolution of magnetization normalized to the mass of the unground samples was presented and discussed in [43]. In our previous study, the process of MWCNT purification by functionalization was observed to cause the iron contamination to drop in these nanotubes by about 90%. This is one of the reasons why the measured magnetization values (*M_S_* and *M_r_*) were significantly lower in the functionalized MWCNTs (-COOH and COONH_4_) compared to the *as prepared* nanotubes.

**Figure 4:**
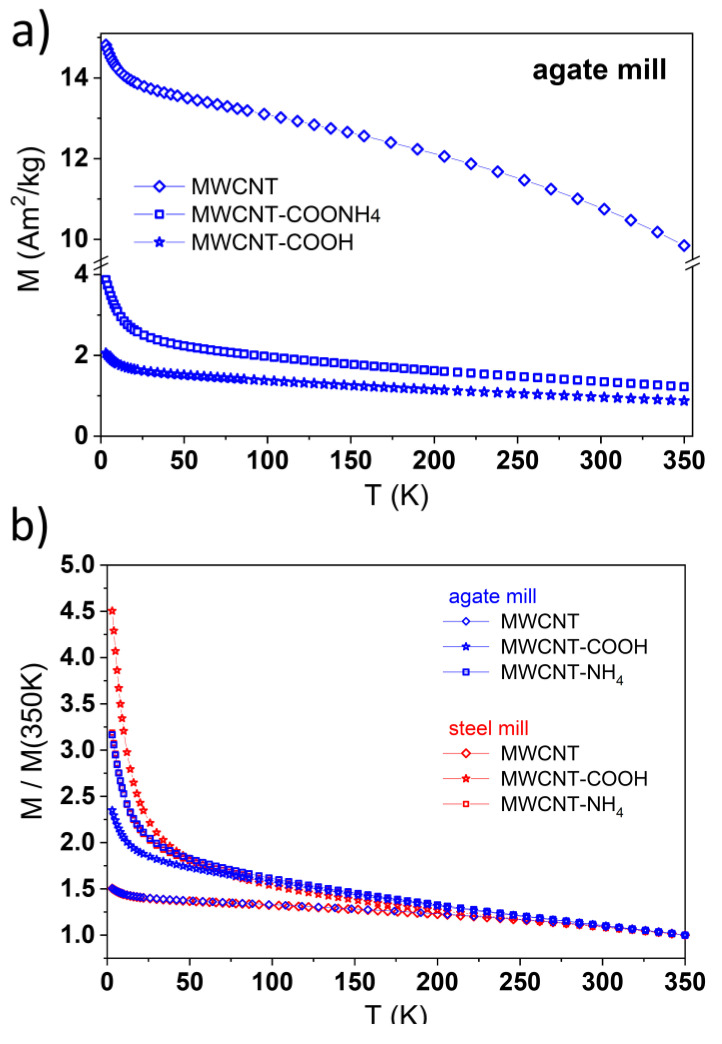
(**a**) Temperature dependences of corrected magnetization (*M*) measured in the field of 4 T for MWCNT (diamonds), and MWCNT-NH_4_ (squares) and MWCNT-COOH (stars) obtained from MWCNTs prepared in agate (squares) mills. (**b**) Magnetization normalized to its value at 350 K measured in the field of 4 T for MWCNT (diamonds), and MWCNT-NH_4_ (squares) and MWCNT-COOH (stars) obtained from MWCNTs prepared in the agate (blue) and steel (red) mill.

In this work, we concentrated on the influence of grinding on the magnetic properties of non-functionalized and functionalized MWCNTs. In Figure 4a, the temperature dependence of carbon-corrected magnetization measured at 3 K for MWCNTs ground with the agate mill is presented. In order to get a better visualization of *M* changes at temperatures below 150 K, in the case of functionalized nanotubes, magnetization values normalized to *M_350_* value at 350 K for each case independently are shown in Figure 4b. This figure contains both sets of data for nanotubes ground in the agate and steel mill. One sees that magnetization depends on the type of the applied mill only in the case of MWCNTs-COOH.

**Figure 5:**
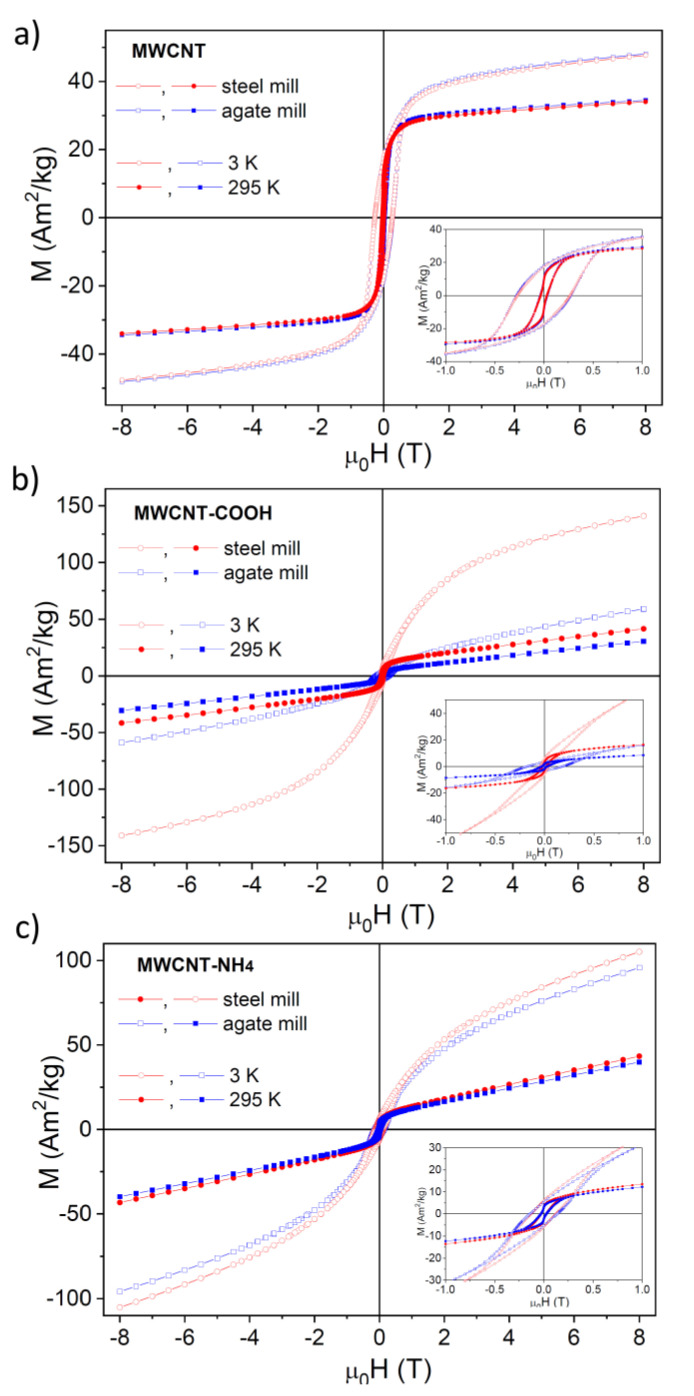
Field dependencies of magnetization (M) for (**a**) *as prepared* MWCNTs, (**b**) MWCNTs-COOH, and (**c**) MWCNTs-COONH_4_ obtained from MWCNTs prepared in the agate (blue squares) and steel (red circles) mill. Empty symbols denote the data measured at 3 K, and full symbols correspond to the data measured at 295 K. The insets show the expansion of the dependencies close to the origin.

The *M* vs. μ_0_*H* hysteresis loops for the non-functionalized and functionalized carbon nanotubes were measured at 3 K and 295 K (Figure 5). Presented values of magnetization were calculated per mass of iron. For *as prepared* nanotubes and nanotubes with NH_4_ groups, both hystereses, regardless of the mill type used, were similar to each other at 3 K and 295 K.

A much larger contribution of a superparamagnetic phase for the MWCNT-COOH sample prepared in the steel mill than that in the agate mill revealed much stronger nonlinearity and larger disparity of the corresponding *M* vs. μ_0_*H* dependences at 3 K.

At 3 K as well as at 295 K, these dependencies did not saturate even at the highest field applied (μ_0_*H* = 8 T). One should also note that the loops for all investigated MWCNTs were characterized by nonzero coercive fields at 295 K, indicating a significant contribution of the ferromagnetic phases even at room temperature. They also showed quite good linear *M*(*H*) dependence for μ_0_*H* fields larger than 4 T at 295 K. Coercive fields (*μ_0_H_c_*) estimated for the carbon nanotubes are shown in Table 2. As one may expect, their values were higher at 3 K than at 295 K. The differences between the coercive fields (Δ*H_c_*) found for unground and ground MWCNTs did not exceed ~10% except for carboxylated nanotubes. In MWCNT-COOH, *μ_0_H_c_* decreased about 4- and 1.5-fold at 3 K and 295 K, respectively, when the steel mill was used (Figure 5, Table 2).

**Figure 6:**
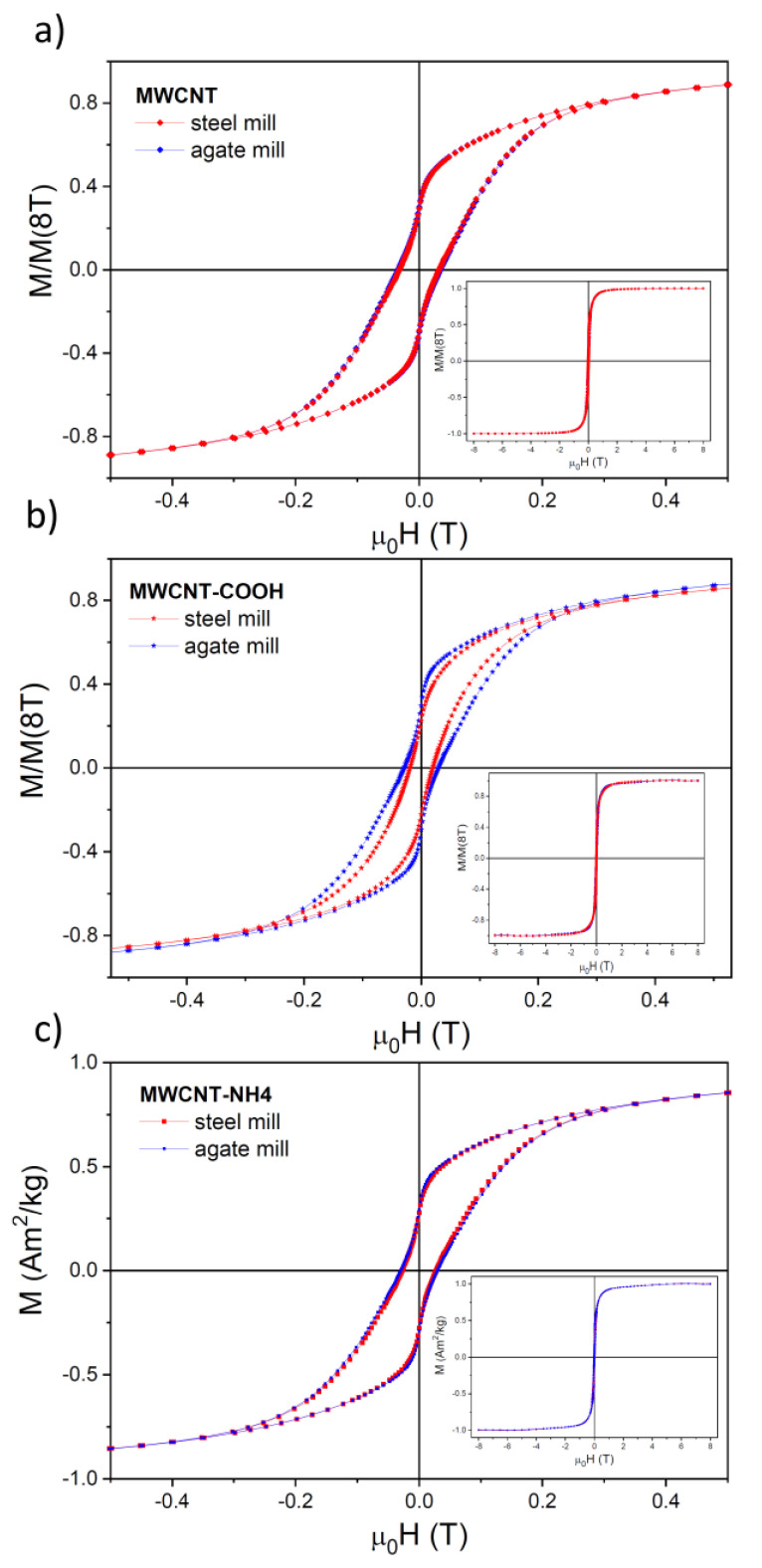
Field dependencies of the normalized magnetization *M/M*(8T) for (**a**) *as prepared* MWCNTs, (**b**) MWCNTs-COOH, and (**c**) MWCNTs-COONH_4_ obtained from MWCNTs prepared in the agate (blue symbols) and steel (red symbols) mill at 295 K after subtracting the corresponding paramagnetic contribution. The insets show the full curves.

Fitting linear dependencies to the *M*(*H*) hysteresis loops at 295 K for |μ_0_*H*| larger than 4 T and subtracting the corresponding paramagnetic contribution from total *M*(*H*) dependencies, one can separate the remaining ferro- and superparamagnetic-like contributions. The corrected *M*(*H*) hysteresis loops at 295 K are shown in Figure 6. One can note clearly a different and nontrivial shape (showing a strong narrowing in the horizontal extent at close to zero *M* values) of the hysteresis loop for all studied nanotubes. There was one exception, MWCNTs-COOH ground in the steel mill. Only for these nanotubes, such narrowing of the hysteresis loop was hardly visible (Figure 6b).

Table 2 also contains the saturation magnetization (*M_s_*) and effective magnetic anisotropy constant (*K_eff_*) determined from the hysteresis loops for *H* >> *H_c_* using the LAS model (the law of approach to saturation) for uniaxial systems [44,45]:

(1) $$\left|M(T,H)\right|=M_{S}(T)-\frac{4K^{2}(T)}{15M_{S}(T)H^{2}}+\left|H\right|\chi_{p}(T)$$

where *K* is the effective anisotropy *K_eff_* and *χ_p_* is the paramagnetic susceptibility (from paramagnetic impurities or superparamagnetism of some fine particles).

The saturation magnetization (expressed per mass of iron) measured at 3 K had higher values for functionalized than non-functionalized MWCNTs and its highest value was found for carboxylated nanotubes triturated in the steel mill (~159 Am^2^/kg). At 295 K, the *M_s_* values were comparable, but those for functionalized nanotubes were still higher than those for non-functionalized nanotubes, but by no more than about 30%. The *M_s_* values given in Table 2 were further used in the calculations of anisotropy constants. The effective anisotropy constant had again higher values for functionalized than for non-functionalized MWCNTs (612 ± 100 kJ/m^3^ vs. 171 ± 18 kJ/m^3^ at 3 K and 311 ± 37 kJ/m^3^ vs. 105 ± 8 kJ/m^3^ at 295 K).

Knowing the saturation magnetization, one may estimate the upper limit for the shape anisotropy constant (*K_d_*) for a spheroidal single domain particle from the dependence:

(2) $$K_{d}=\frac{\mu_{0}M_{s}{}^{2}}{2}(N_{c}-N_{a})$$

where *N_c_* and *N_a_* are the demagnetization factors in the mutually perpendicular directions of the principal spheroid axes. For the limit of the extremely flat (oblate) spheroid, this results in:

(3) $$K_{d}=\frac{\mu_{0}M_{s}{}^{2}}{2}$$

whereas for the extremely elongated (prolate) spheroid, which is more appropriate for the present case, [46,47]:

(4) $$K_{d}=\frac{\mu_{0}M_{s}{}^{2}}{4}$$

They are several times lower than the K_eff_ values found for both ”*as preapared*” and functionalized nanotubes (Table 2).

The temperature dependence of saturation magnetization for the small iron-enriched inclusions in carbon nanotubes can be described by the semi-empiric dependence:

(5) $$M_{s}(T)=M_{s}^{}(0)(1-{\left(T/T_{0S}\right)}^{\alpha })$$

where 1.65<α<2.6 and (*T*_0*s*_) is a characteristic temperature above which magnetic ordering disappears [52,53]. In our estimations, we used α=1.8, which is adequate for small iron domains and nanoparticles [53]. The estimated average value *T*_0*s*_ = 472 ± 92 K. Taking into account only functionalized nanotubes, one gets *T*_0*s*_ = 412 ± 28 K. For non-interacting single-domain nanoparticle assembly [54], which is not affected by superparamagnetic fluctuation there is proportionality between $M_{s}(T)/M_{s}(0)$ and $M_{r}(T)/M_{r}(0)$. Therefore, one can expect that the equation should also express the temperature relationship of remanence, [52,53]:

(6) $$M_{r}(T)=M_{r}(0)(1-{\left(T/T_{0r}\right)}^{\alpha })$$

In this case, the average value of the *T_0r_* parameter calculated for all MWCNT’s is about 431 ± 11 K taking into account α=1.8.

### 3.3. TEM Images

**Figure 7:**
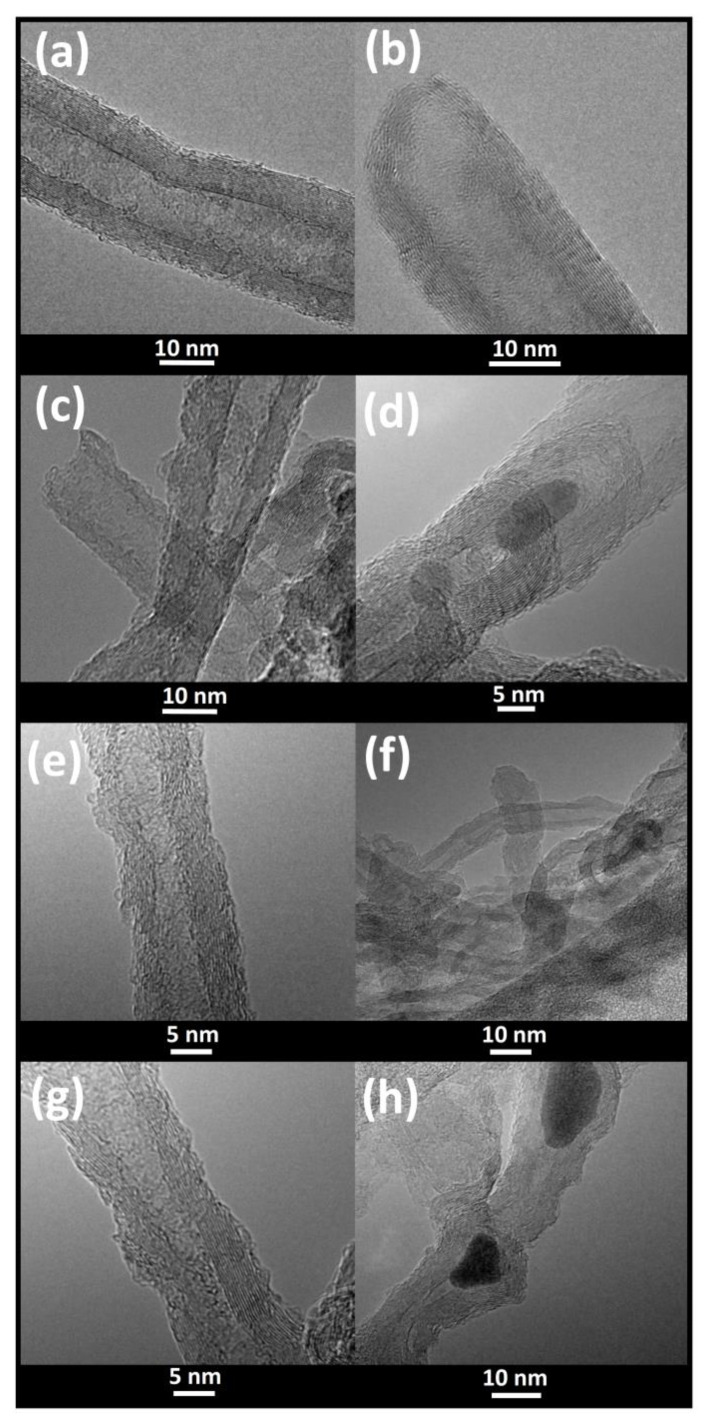
Example TEM images of high resolution for MWCNTs-COOH (**a**), MWCNTs-COONH_4_ (**b**), MWCNTs-COONH_4_ ground with the agate mill (**c**,**d**), and MWCNTs-COOH ground with the agate mill (**e**,**f**) and the steel mill (**g**,**h**). In (**d**) and (**h**), large Fe-nanoparticle built-in MWCNTs are visible. Some small metallic nanoparticles bound within the walls of carbon nanotubes are visible as dark spots (**e**,**g**).

In Figure 7, examples of high-resolution images of functionalized MWCNTs, ground in the agate and steel mill, are shown. Their outer and inner surfaces are no longer smooth, as in unmilled nanotubes (Figure 7a,b; [43]). One can also observe the fracture sites of several walls at once (Figure 7d,e,g).

One sees that iron nanoparticles are located within the structure of MWCNTs, in the empty space between their walls. Their sizes are usually ~1 nm. However, when they are embedded inside the nanotube (Figure 7d,h), Fe-NPs can form much larger structures with a diameter even >10 nm. These Fe nanoparticles may be spherical or oval, but the latter ones are more abundant. EDX experiments show that the nanoparticles closed inside MWCNTs contain mainly Fe and C atoms [43]. However, in the case of carboxylated CNTs triturated in the steel mill, the nanoparticles may also contain a significant amount of oxygen in iron clusters (Figure S4), which is consistent with the results from the Mössbauer experiments.

## 4. Discussion

In order to design new materials based on carbon nanotubes (CNTs) one has to know their chemical and physical properties. Iron complexes interacting with the CNT composite matrix may significantly modify the electric and magnetic characteristics of the nanotubes [9,14,15,16,17,18,20,43]. Iron contamination is always present in CNTs when Fe is used as a catalyst during their preparation. Moreover, it has been already shown that functionalization, which is often used as a method of CNT purification, changes the redox and structural properties of iron aggregates embedded inside MWCNTs but cannot remove entirely metal compounds [55,56]. In the case of *as prepared* MWCNTs studied by us, cementite had the main contribution, and only a small amount of α–Fe was detected. We also observed mixed spin states of Fe^2+/3+^ in oxides/hydroxides and/or ferrihydrates (paramagnetic or magnetically ordered in the case of CNTs ground in the agate mill). We also detected high spin Fe^2+^ in Fe_x_O phases. Carboxylation and then adding ammonium groups modify the magnetic properties of MWCNTs and may cause a loss of up to 90% of the iron content in MWCNTs [43]. Nevertheless, Mössbauer spectroscopy and VMS are sensitive methods to monitor the iron contaminations in these carbon nanotubes. These various Fe-phases were still present in functionalized nanotubes, albeit in a different ratio, but Fe_3_C always had the highest contribution, with the exception of carboxylated nanotubes ground in the steel mill (Figure 3). Interestingly, these iron complexes formed in our MWCNTs during their growth differ from those reported for Fe-filled CNTs [18,57,58], when pyrolyzing a mixture of ferrocene and C_60_ in the Ar atmosphere led to the formation of α–Fe, γ–Fe, and Fe_3_C in CNTs. For such systems, a model of an elongated core consisting of α–Fe particles, surrounded by a γ–Fe shell coated with a layer of cementite, was proposed [21,59]. There was also a relative increase in the γ–Fe phase content at the apex of the nanotube. [18,21]. Only α-Fe, γ-Fe, and Fe_3_C were observed in MWCNTs grown by chemical vapour deposition with ferrocene as a precursor [60,61]. The relative ratio of the different iron compounds depended on the sample preparation.

Our experimental data presented in this work show that mechanical treatment can cause damage to the outer walls of nanotubes or even fractures and dislocation of their internal walls (Figure 7). It can also affect the iron phases embedded in MWCNTs (Figure 1 and Figure 2, Figures S1 and S2, Tables S1–S3). Trituration of CNTs either in the agate mill or in the steel mill resulted in a decrease in the content of the high spin ferrous state in the magnetically ordered Fe_x_O phase and in the paramagnetic phase as well as an increase in the α-Fe phase in the *as prepared* and ammonium nanotubes. Hyperfine parameters of the magnetic Fe_x_C_y_ fraction with a low hyperfine field of about 14 T observed in unground MCNTs-COONH_4_ were similar to those reported for the C:Fe ratio of about 0.4 ÷ 0.6 with about four carbon atoms in the Fe neighborhood [37]. This phase disappeared in the ground ammonium nanotubes. The most significant modification of the iron compounds inside MWCNTs was found for MWCNTs-COOH ground in the steel mill. We observed a 4-fold decrease in the Fe_3_C fraction and a simultaneous formation of magnetic iron-oxide phases having the highest contribution at 85 K (up to about 42%) as well as a 2-fold increase in the α-Fe content. As judged from the Mössbauer spectrum, about 50% of the Fe-oxides fraction showed superparamagnetic behavior at RT. Together with the high hyperfine magnetic field of 55 T at low temperature, this indicates that the superparamagnetic nanoparticles were composed of Fe_2_O_3_ and were probably smaller than 5 nm [36,62,63].

TEM images demonstrated that iron complexes were localized and present in nanotubes as inclusions to form nanoparticles (NPs) of different dimensions. The size of the smallest NPs located between the walls of MWCNTs did not exceed ~1 nm, but the dimension of those trapped inside the nanotube channel was about 5–10 nm and even >10 nm. (Figure 7, Figure S4 and [43]). Because these NPs were separated (non-interacting), their size and shape should determine the magnetic behavior of MWCNTs in an external magnetic field [64].

### 4.1. Characterization of Magnetic Properties of Fe-NPs Embedded Inside the Carbon Matrix of MWCNTs

There are some distinct characteristic magnetization and magnetic behavior regimes of NPs depending on their size [19,65,66]. Below a critical diameter (*d_c_*), NPs become a single domain, where the *d_c_* value in the range 10 ÷ 100 nm depends both on the material and geometrical properties of NPs. With further dimension reduction, the thermal fluctuation energy may be enough to overcome the anisotropy barrier, and NPs become superparamagnetic: magnetization fluctuates along the easy magnetization axis, and there is no hysteresis. Superparamagnetic fluctuations become suppressed below the blocking temperature *T_B_*, and hysteretic magnetization is observed for single domain NPs with diameters above a threshold diameter *d_t_* that depends on the sample temperature.

The particle shape and matrix in which magnetic NPs are enclosed have a big impact on the magnetic properties of the whole material [27,65,67,68]. One may determine the size of stable single domains based on the theories developed for fine particles and bulk systems [69,70]. Knowing the saturation magnetization of bulk cementite (*M_S_* ~169 Am^2^/kg at a temperature close to 0 K [71] and ~136 Am^2^/kg at 293 K) [72], we calculated the magnetostatic exchange length (in SI units):

(7) $$l_{lex}=\sqrt{\frac{2A}{\mu_{0}M_{S}{}^{2}}}=\sqrt{\frac{A}{K_{d}}}$$

where *A* is the exchange-stiffness constant and *K_d_* is the maximum demagnetization energy density. A critical size/radius for a cylinder and sphere can be calculated using the relation *R_critcyl_* ≈ 2.6*l_ex_* and *R_critsp_* ≈ 3.6*l_ex_*, respectively [70,73,74]. A typical *A* value at temperatures close to 0 K is ~10^−10^ J/m [75], and at room temperature, it can be approximated from the relation:

(8) $$\frac{A(T)}{A(0)}={\left(\frac{M_{S}(T)}{M_{S}(0)}\right)}^{\alpha }$$

where α = 1.75 [76,77]. This power is more appropriate for *T* ≤ 0.5 × *T_C_* (*T_C_*—Curie temperature). We used *α* = 1.8 as mentioned above [53] in the evaluation of the exchange stiffness constant at a higher temperature *A(T)* and then the effective anisotropy length *l_Keff_* and the magnetostatic exchange length *l_ex_* (see Table 3). The *T_C_* temperature reported for bulk cementite is about 483 K [78,79], and the characteristic temperature at which magnetic ordering disappeared in our Fe_3_C-NPs was about 430 K. Therefore, in our calculations of *l_ex_* and *l_K_* (defined below) for bulk Fe_3_C, we used *A(T)* = 6.8 × 10^−11^ J/m at 295 K. The estimated *l_lex_* values were about 9.8 nm (*d_c_* ≈ 51 ÷ 71 nm) and 10.1 nm (*d_c_* ≈ 52 ÷ 73 nm) at the low and high temperature, respectively. Using reported values of the magnetocrystalline anisotropy energy (*K*) of about 334 kJ/m^3^ and 150 kJ/m^3^ at 5 K and 300 K, respectively, for bulk Fe_3_C [80], we obtained the crystalline anisotropy length

(9) $$l_{K}=\sqrt{\frac{A}{K}}$$

to be about 17 nm (*d_c_* ≈ 88 ÷ 122 nm) at the low temperature and about 21 nm (*d_c_* ≈ 109 ÷ 151 nm) at room temperature. The characteristic *d_t_* diameter can be estimated under conditions where the energy of magnetization reversal is equal to the thermal energy [69]. It was about 5.2 nm (for *K_d_*) and 6.0 nm (for *K*) at 295 K. At 3 K, this parameter was ~1 nm for both anisotropy constants.

Magnetostatic exchange length and anisotropy length (*l_Keff_*) for NPs enclosed inside the investigated carbon nanotubes are shown in Table 3. They were calculated from the formula given above, but instead of the magnetocrystalline anisotropy constant *K*, the effective anisotropy constant *K_eff_* (this work, Table 2) was used. The exchange-stiffness constant for NPs was evaluated according to the relation

(10) $$\frac{A_{dill\_NPs}(T)}{A_{non\_dill\_bulk}(T)}=\frac{M_{S}{}_{dill\_NPs}(T)}{M_{S}{}_{non\_dill\_bulk}(T)}$$

for diluted systems [82]. Applied values of *M_s_* for iron NPs are given in Table 2.

Considering Mørup and Topsøe’s mechanism [83] one may obtain a diameter of the iron nanoparticles responsible for the magnetic ordering observed in Mössbauer experiments [62]. The values of *H_hf_* decrease with increasing temperature due to the collective magnetic excitations. In their model, the hyperfine field *H_hf_* for a particle of volume *V* at a temperature *T* is given by:

(11) $$H_{hf}(V,T)=H_{hf}(0^{o}K)\left[1-\frac{k_{B}T}{2K_{eff}V}\right]$$

for *k_B_T << K_eff_V*, where *k_B_T* is the thermal energy and *K_eff_* is the effective anisotropy constant. In Table 3, the calculated diameters (*d_SM_*), according to this formula using estimated values *K_eff_* (Table 2), are shown. The average diameter estimated for *as prepared* and functionalized nanotubes was about 10.1 ± 1.1 nm, 8.6 ± 0.6 nm, respectively.

One sees that the estimated *d_SM_* diameters were comparable to effective anisotropy length *l_Keff_* found for the iron NPs embedded in the investigated MWCNTs at both temperatures, 3 K and 295 K. For our spheroidal NPs, we estimated the limits of the *l_ex_* parameter for prolate and oblate particles (Table 3). They were about 1.2 ÷ 4-fold larger than the *l_Keff_* values obtained for corresponding CNTs. The higher ratio *l_ex_/l_Keff_* was observed for functionalized nanotubes at 295 K.

If there are multiple barriers, the one with the shortest characteristic length determines the properties of the material [84]. In the case of bulk cementite *l_K_ > l_ex_* but for our Fe_(C,O)_-NPs, *l_Keff_* < *l_K_* and *l_ex_* > *l_Keff_*. This suggests that in bulk cementite, the magnetostatic energy barrier is higher than the crystalline anisotropy energy barrier, whereas in the case of Fe_(C,O)_-NPs trapped inside carbon nanotubes, the opposite is true. Therefore, in MWCNTs, the effective anisotropy constant of Fe_(C,O)_-NPs was the most important parameter as one expected. In all our samples, *K_eff_* was significantly higher than *K_d_* and it was higher for functionalized than for non-functionalized nanotubes. This means that the main contribution to K*_eff_* was from uniaxial magnetic anisotropy, which can be altered by surface interactions as a result of the entrapment of magnetic NPs in carbon nanotubes and the functionalization. In elongated NPs, the anisotropy shape determines their axis of easy magnetization along their long axis, but this effect may be less pronounced in nanoparticles with a small size ratio, and this is our case. In addition, the covalent interactions of Fe_(C,O)_-NPs with the carbon matrix of the non-functionalized nanotubes can change the surface anisotropy, which may have an impact on both uniaxial and shape anisotropy constants. It was estimated that about 30% of atoms forming a 10-nm NP are surface atoms [48]. Those atoms on the contact surface between nanoparticles and the inner side of nanotubes can modify the distribution of magnetic poles at the edge of ferromagnetic NPs. One cannot also exclude that in a case of a charge transfer, weak magnetic poles can be additionally induced within the carbon matrix [85,86]. Especially during milling, stimulated diffusion of atoms accompanied by chemical reactions and an increased magnetic frustration on the contact surface may occur. Therefore, we think that surface anisotropy can have a significant contribution to *K_eff_*. Indeed, we observed a 2.5 ÷ 4-fold increase in *K_eff_* for functionalized MWCNTs compared to their non-functionalized counterparts. Changes in the strength of covalent interactions between Fe_(C, O)_-NPs and the surface of the carbon nanotube and in the NP surface exposed to the free space inside the nanotube as a result of CNT functionalization may explain the increase in the *K_eff_* value in carboxylated and ammonium MWCNTs as well as a strong impact of functionalization on the increase of *K_d_*. Another effect, which may be associated with changed interactions in the interface between the carbon matrix and the edge of nanoparticles, may be the dilution of the concentration of Fe atoms and the change in their chemical state in the outer part of NPs.

The surface atoms generally significantly contribute to the total magnetic moments of nanoparticles due to the large surface to volume ratios [48]. This effect was observed here for MWCNTs containing Fe_(C,O)_-NPs. The differences in *M_S_* between functionalized and non-functionalized nanotubes are related to the amount and size of incorporated iron nanoparticles as well as their interactions with the modified inner surface of MWCNTs containing COOH and COONH_4_ groups. These factors have an impact on the coercivity and remanence of the materials.

The *M_r_* and *H_c_* parameters (see Table 2) found for Fe_3_C NPs in *as prepared* MWCNTs (ground and unground) were similar to those obtained for synthesized Fe_3_C NPs of comparable size to ours [87]. Synthesized Fe_3_C nanoparticles in the carbon matrix had average diameters of about 12 ± 5 nm and showed a finite coercivity in the range 0.024 ÷ 0.036 T and the saturation magnetization between 8 and 15 Am^2^/kg at room temperature. Their coercivity increased to about 0.25 T at 10 K. The remanence-to-saturation ratio of *M_r_/M_S_* changed from about 0.32 to 0.5 as the temperature increased. In our non-functionalized nanotubes, the *M_r_/M_S_* ratio was slightly smaller, and it showed a weaker dependence on the temperature (Table 2). A ratio of 0.5 was predicted for an assembly of non-interacting, randomly oriented single domain nanoparticles [54,87]. The MWCNTs system created a matrix that spatially isolates Fe(C,O)-NPs and prevented their interactions (Figure 7). However, in the case of functionalized nanotubes, *M_r_/M_S_ << 0.5*. The ratio varied between about 0.04 and 0.07 (Table 2). The lowest value of *M_r_/M_S_* was found for MWCNTs-COOH ground in the steel mill at 3 K. In general, the functionalization of MWCNTs with carboxyl and ammonium groups resulted in a 2-fold decrease in *H_c_* at 3 K and a 3-fold decrease in *M_r_* at both temperatures. The increase in *K_eff_* correlated with an increase in *K_d_* and *M_S_*, and the values of these parameters increased with the series as follows: *as prepared* MWCNTs < MWCNTS-COOH < MWCNTs-COONH_4_. At the same time, both anisotropy constants increased as the *H_c_* field decreased.

The coercive fields observed at the room temperature for the investigated MWMCNTs were comparable or even 2-times higher than those found in Fe_3_C nanoparticles with higher diameters, in Fe-filled MWCNTs and Fe-SWCNTs or in iron carbide NPs embedded in a carbon matrix [18,61,87,88,89]. The a*s prepared* MWCNTs had *M_r_* and *H_c_* values the same as cobalt ferrite nanomagnets encapsulated inside the CNTs [90].

### 4.2. Unique Effects of Milling on the Magnetic Properties of MWCNTs-COOH

More detailed testing of *H_c_*, *M_r,_* and *M_s_* parameters revealed a unique effect of milling on the magnetic properties of MWCNTs-COOH, which additionally depends on the type of mill used. When the agate mill was applied, *M_r_* decreased 2-fold compared to the carboxylated nanotubes unground and ground in the steel mill at respective temperatures. Only a slight decrease in the *K_eff_* anisotropy constant and *M_s_* magnetization (in both cases by ~16%) at 3 K accompanied this effect, but these differences disappeared at 295 K (Table 2). These differences seem insufficient to explain the detected *M_r_* decrease in these carboxylated CNTs. Therefore, we believe that some specific chemical modifications in the carbon matrix on the contact surface with iron NPs result in changes in their dynamic properties. However, this suggestion needs to be confirmed in the future.

Another very interesting effect we observed for MWCNTs-COOH ground in the steel mill was the coercive field that was 4- and 1.4-times lower than in the other functionalized (ground and unground) samples at 3 K and 295 K, respectively. Remanence remained unchanged, but the saturation magnetization increased about 2-fold at 3 K compared to the carboxylated nanotubes unground and ground in the agate mill. At the same time, *K_d_* increased more than 2-fold, while *K_eff_* changed by less than 30%. These differences diminished at room temperature. Thus, all parameters characterizing the carboxylated nanotubes ground in the steel mill were similar to those found for the ammonium CNTs at 295 K except of coercivity. Without doubt, the transfer of almost 50% of the Fe_3_C phase mainly into the superparamagnetic Fe_2_O_3_ phase was responsible for the phenomenon. Cementite belongs to soft magnetic materials, but α-Fe and iron oxides are softer magnets [48].

In MWCNTs-COOH ground in the steel mill, iron oxide phases formed due to the specific conditions during the trituration. Most likely, friction and temperature were the main factors stimulating the chemical changes in iron embedded inside MWCNTs-COOH. The only explanation of the obtained results is to assume that H^+^ can be easily adsorbed from the carboxyl group by the steel surface, enabling the reaction of COO^ࢤ^ with the encapsulated iron. It cannot be excluded that α-Fe in the carbon nanotubes is the intermediate state in the reaction chain [91,92]. This suggestion is in line with the known high affinity of steel for hydrogen [93,94]. Therefore, this effect was absent in *as prepared* MWCNTs and MWCNTs-COONH_4_ as well as MWCNTs-COOH when the agate mill was used. It should be noted that in the functionalized nanotubes, which are opened, their inner part is also functionalized due to their capillarity and wetting [84]. Moreover, it should be borne in mind that the accessible surface of iron nanoparticles inside the channel of CNTs was also functionalized.

The rigid carbon matrix of nanotubes keeps nanoparticles in a fixed position. Thus, the shape of hysteresis loops (Figure 5) can be attributed to the superposition of two hysteresis loops, one arising from the more perpendicular and the other form the more parallel orientation of the easy axis of magnetization of NPs to the direction of the external magnetic field [18]. Another possible explanation includes antiferromagnetic coupling between the surface of embedded NPs and the inner surface of nanotubes, but this needs further work, which is in progress. The disappearance of this effect in carboxylated CNTs ground in the steel may be associated with a more random distribution of the easy magnetization axis due to the chemical changes within the interface between NPs and the interior of the nanotube.

## 5. Conclusions

In this paper, we examined commercially available *as prepared* MWCNTs and their two functionalized forms containing -COOH and –COONH_4_ groups in terms of carbon nanotubes magnetic properties. Functionalization caused a release of a significant amount of iron impurities. The Fe_3_C phase, which is responsible for MWCNTs magnetism, dominated in the NPs embedded inside the nanotubes. In this work, we focused on the study of magnetic properties of MWCNTs triturated in a ball mill. We used two types of grinding balls and cylinders: agate and steel.

The grinding process significantly increased the amount of crushed MWCNTs external and internal walls, increasing their porosity. Fe_(C,O)_-NPs was also highly affected. Mechanical grinding of studied MWCNTs did not further remove iron but changed its phases, both their individual content and chemical composition. In ground *as prepared* and functionalized MWCNTs, Fe_3_C remained the dominant iron phase in Fe-NPs at a similar level to that of the corresponding unground nanotubes. However, there was one exception—MWCNTs-COOH triturated in the steel mill. The cementite fraction decreased more than 4-fold in these nanotubes with the simultaneous enhanced formation of superparamagnetic iron oxides and an increase in the α-Fe fraction, confirming the role of -COOH groups in the Fe_3_C phase transition.

We proved that Fe_(C,O)_-NPs embedded inside MWCNTs are responsible for their magnetic properties as single domain non-interacting particles with diameters of about 10 nm. The milling process revealed a unique impact of carboxyl groups (-COOH), in contrast to carboxylic ammonia groups (-COONH_4_), on the coercive and remanent field in functionalized nanotubes. The values of M_S_, Mr, and H_c_ were 37.1 ± 1.5 Am^2^/kg (49 ± 6 Am^2^/kg), 8.9 ± 0.3 Am^2^/kg (2.6 ± 0.5 Am^2^/kg), and 32 ± 1 mT (26 ± 4 mT), respectively, at 295 K for NPs embedded in *as prepared* (functionalized) MWCNTs.

Our observations may help to develop simple and cheap methods for MWCNT functionalization with the intention of obtaining desired chemical and magnetic properties. In particular, the procedure for grinding carboxylated CNTs with embedded iron nanoparticles using a steel mill may be used in the production of high-performance microwave absorption materials based on CNTs and magnetic nanocomposites for medical, environmental or military applications. For example, one of the important applications of carbon nanotubes, recognized in recent years, is the production of paints and varnishes conducting electric current. Surfaces painted with this type of paint have microwave screening capabilities in the GHz and THz range. Electromagnetic interface (EMI) barriers can remove the ubiquitous electromagnetic smog, which may have an impact on working electronic systems.

## Figures and Tables

**Table 1:** Properties of the grinding jars and balls.

Grinding Jar Material	Agate	Stainless Steel
Volume	250 mL	500 mL
Inner diameter	76 mm	100 mm
Designation	SiO2	X90CrMoV18
Hardness	6.5–7.0 Mohs	265 HB
Tensile strength	−	≤925 N/mm^2^
Density	2.65 g/mL	7.7 g/mL
Grinding Ball material	Agate	Stainless Steel
Grinding Ball diameter	10 mm	10 mm

**Table 2:** Coercive and remanent fields (*H_c_* and *M_r_*), saturation magnetization (*M_S_*), effective anisotropy constant (*K_eff_*), and shape anisotropy constant (*K_d_*) for *as prepared* MWCNTs, MWCNTs-COOH, and MWCNTs-COONH_4_ unground and ground in the agate and steel mill.

	MWCNTs	*H_c_* [T]	*M_r_* [Am^2^/kg Fe]	*M_s_*^1^ [Am^2^/kg Fe]	*M_r_/M_s_*	*K_eff_*^1^ [kJ/m^3^]	*K_d_*^2^ [kJ/m^3^]
control	3 K						
	*as prepared*	0.282	19.6	55	0.359	192	54 ÷ 108
	-COOH	0.176	6.0	89	0.067	538	145 ÷ 290
	-COONH_4_	0.124	4.6	114	0.040	682	235 ÷ 471 (172 ÷ 344)
	295 K						
	*as prepared*	0.032	9.1	39	0.233	114	27 ÷ 55
	-COOH	0.029	3.1	42	0.074	290	32 ÷ 63
	-COONH_4_	0.027	2.3	51	0.044	380	48 ÷ 96 (35 ÷ 70)
agate mill	3 K						
	*as prepared*	0.276	18.1	51	0.358	159	46 ÷ 93
	-COOH	0.180	3.4	75	0.045	450	102 ÷ 203
	-COONH_4_	0.140	5.3	124	0.043	701	280 ÷ 560 (205 ÷ 410)
	295 K						
	*as prepared*	0.034	8.5	36	0.236	104	24 ÷ 48
	-COOH	0.027	1.7	41	0.041	289	30 ÷ 60
	-COONH_4_	0.028	2.6	55	0.048	277	55 ÷ 111 (40 ÷ 81)
steel mill	3 K						
	*as prepared*	0.268	17.4	51	0.343	162	47 ÷ 94
	-COOH	0.045	5.9	159	0.037	686	460 ÷ 920 (336 ÷ 673)
	-COONH_4_	0.127	6.0	114	0.053	616	234 ÷ 468 (171 ÷ 342)
	295 K						
	*as prepared*	0.032	9.0	36	0.249	98	24 ÷ 48
	-COOH	0.019	3.1	53	0.059	313	51 ÷ 101 (37 ÷ 74)
	-COONH_4_	0.026	2.8	51	0.056	317	47 ÷ 93 (34 ÷ 68)

^1^. The saturation magnetization (*M_s_*) and effective magnetic anisotropy constant (*K_eff_*) determined from the hysteresis loops for *H* >> *H_c_* using the LAS model (the law of approach to saturation) for uniaxial systems [44,45]. *M_r_* and *M_s_* values were calculated on the mass of iron contained in the corresponding carbon nanotubes. ^2^. Upper limit of the shape anisotropy (*K_d_*) estimated from the dependence $K_{d}=\frac{\mu_{0}M_{s}{}^{2}}{2}$, which is valid for non-interacting particles having an oblate spheroidal shape (b >> a) [46,48] and lower limit calculated for prolate nanoparticles $K_{d}=\frac{\mu_{0}M_{s}{}^{2}}{4}$ [47]. Density for cementite: 7.6 g/cm^3^ [49,50]^.^ In parentheses, *K_d_* values are given for weighted average density: 6.5 g/cm^3^; in this case 5.3 g/cm^3^ for Fe_2_O_3_, ~4.0 g/cm^3^ for ferrihydrates [51], 7.9 g/cm^3^ for Fe, and 7.6 g/cm^3^ for Fe_3_C. Contributions of these iron phases were taken from the Mössbauer spectra at 85K (Table S1).

**Table 3:** Diameters (*d_SM_*) of Fe_(C,O)_-NPs embedded in *as prepared* MWCNTs, MWCNTs-COOH, and MWCNTs-COONH_4_ unground and ground in the agate and steel mill determined on the basis of hyperfine fields obtained in Mössbauer experiments and the effective anisotropy constant. Magnetostatic exchange lengths (*l_ex_*) and anisotropy lengths (*l_Keff_*) were calculated from *A* parameters estimated for NPs. See the text for details and description below the table.

	MWCNTs	*d_SM_* [nm]	*l_Keff_* [nm]	*l_ex_* [nm]	*l_Keff_* [nm]	*l_ex_* [nm]
control			3 K		295 K	
	as prepared	11.3	13.0	17.3 ÷ 24.4	9.6 12.0 *	17.9 ÷ 25.3 22.5 ÷ 31.8 *
	-COOH	9.3	9.9	13.5 ÷ 19.1	5.0 7.4 *	14.6 ÷ 20.6 21.7 ÷ 30.7 *
	-COONH_4_	9.2	9.9	12.0 ÷ 16.9	4.9 7.3 *	13.0 ÷ 18.3 19.6 ÷ 27.6 *
agate mill			3 K		295 K	
	as prepared	9.1	13.7	18.0 ÷ 25.4	12.5 15.7 *	18.6 ÷ 26.3 23.3 ÷ 33.0 *
	-COOH	8.2	9.9	14.8 ÷ 20.9	7.2 10.0 *	15.7 ÷ 22.2 22.0 ÷ 31.1 *
	-COONH_4_	8.1	10.2	11.4 ÷ 16.2 (13.4 ÷ 18.9)	7.8 11.9 *	12.4 ÷ 17.6 (14.5 ÷ 20.5) 18.9 ÷ 26.7 * (22.1 ÷ 31.2 *)
steel mill			3 K		295 K	
	as prepared	9.8	13.6	17.9 ÷ 25.3	12.9 16.3 *	18.5 ÷ 26.2 23.3 ÷ 33.0 *
	-COOH	8.0	11.7	10.1 ÷ 14.3 (11.8 ÷ 16.7)	6.4 11.0 *	11.3 ÷ 16.0 (13.2 ÷ 18.7) 19.3 ÷ 27.3 * (22.5 ÷ 31.9) *
	-COONH_4_	8.9	10.5	12.1 ÷ 17.0 (14.1 ÷ 20.0)	7.1 10.7 *	13.1 ÷ 18.5 (15.3 ÷ 21.6) 19.7 ÷ 27.9 * (23.1 ÷ 32.6) *

* These values were estimated taken into account *A_NPs_(T)* calculated using *A_bulk Fe3C_ (T)* and *M_S_bulk_Fe3C_*(T). The remaining *l_ex_* and *l_Keff_* were assessed based on an approach in which the *A_NPs_(T)* values were calculated from: $\frac{A_{NPs}(T)}{A_{NPs}(0)}={\left(\frac{M_{S}\left(T\right)\_NPs}{M_{S}{\_}_{NPs}(0)}\right)}^{\alpha }$ for α = 1.8, which is adequate for small domains and particles [53,76,77]. *A_NPs_(0)* was calculated using *A_bulk Fe3C_(0)* and *M_S_bulk_Fe3C_(0)* values. The values in parentheses were estimated for the weighted average density of the magnetic Fe phases (6.5 g/cm^3^, details are given in Table 2). In our estimations of *d_MS_* parameters, we took into account the hyperfine magnetic field changes due to the Debye temperature [81].

## Data Availability

Data is contained within the article or Supplementary Materials.

## Supplementary Materials

The following are available online at https://www.mdpi.com/article/10.3390/ma14144057/s1, Table S1: Hyperfine parameters fitted to the Mössbauer spectra measured at 85 K (*IS*—isomer shift related to the metallic Fe, *QS*—quadrupole splitting, *H_hf_*—hyperfine magnetic field, ∆*Q*—quadrupole splitting distribution, ∆*H*—magnetic field distribution, C—relative contribution, *Γ*—line width), Figure S1: Mössbauer spectra for (a) *as prepared* MWCNTs, (b) MWCNTs-COOH, and (c) MWCNTs-COONH_4_: left column—the control group, middle column—after using the agate mill, right column—after using the steel mill, measured at 220 K, Table S2: Hyperfine parameters fitted to the Mössbauer spectra measured at 220 K (*IS*—isomer shift related to the metallic Fe, *QS*—quadrupole splitting, *H_hf_*—hyperfine magnetic field, ∆*Q*—quadrupole splitting distribution, ∆*H*—magnetic field distribution, *C* —relative contribution, *Γ*—line width), Figure S2: Mössbauer spectra for (a) *as prepared* MWCNTs, (b) MWCNTs-COOH, and (c) MWCNTs-COONH_4_: left column—the control group, middle column —after using the agate mill, right column—after using the steel mill, measured at 295 K, Table S3: Hyperfine parameters fitted to the Mössbauer spectra measured at 295 K (*IS*—isomer shift related to the metallic Fe, *QS*—quadrupole splitting, *H_hf_*—hyperfine magnetic field, ∆*Q*—quadrupole splitting distribution, ∆*H*—magnetic field distribution, *C*—relative contribution, *Γ*—line width), Table S4: Metal and semimetal concentrations of MWCNTs obtained by use of ICP-MS method. Concentrations are given in [μg/g]. n.d. —below the detection limit, Figure S3: Temperature dependencies of the magnetic moment (μ) measured in the field of 4 T for MWCNTs-COOH obtained from MWCNTs prepared in the agate (blue squares) and steel (red circles) mill. Empty symbols denote the data as measured, and full symbols correspond to the values corrected for the carbon contribution, Figure S4: TEM image of a large nanoparticle in MWCNTs-COOH and experimental evidence (EDX measurements) that it contains Fe, C, and O is given.

## Nomenclature

CNTs	carbon nanotubes
MWCNTs	multiwall carbon nanotubes
SWCNTs	singlewall carbon nanotubes
Fe-NPs	iron nanoparticles
MAMs	microwave absorption materials
VSM	vibrating sample magnetometer
VSM	vibrating sample magnetometer
PPMS	physical property measurement system
TEM	transmission electron microscope
EDX	energy dispersive X-ray spectroscopy
ICP-OES	inductively coupled plasma—optical emission spectrometer
MD method	microwave digestion method
LAS	law of approach to saturation
EMI	electromagnetic interface
IS	isomer shift related to the metallic Fe [mm/s]
QS	quadrupole splitting [mm/s]
H_hf_	hyperfine magnetic field [T]
∆*Q*	quadrupole splitting distribution [mm/s]
∆*H*	magnetic field distribution [T]
C	relative contribution [%]
Γ	line width [mm/s]
Γ	line width [mm/s]
IS	isomer shift related to the metallic Fe [mm/s]
d_SM_	diameter of NPs estimated from Mössbauer data
Vk_B_	volume of a particle [m^3^]Boltzmann constant, 1.380649x10^-23^ J/K
M	mass magnetization [Am^2^/kg]
M_S_	saturation magnetization [Am^2^/kg]
M_r_	remanence, remanent field [Am^2^/kg]
μ_0_H_c_	coercivity, coercive field [T]
ΔH_c_	differences between the coercive fields [T]
μ_0_*H*	external field [T]
μ_0_	vacuum magnetic permeability, 4π × 10^−7^ H/m
μ	magnetic moment [Am^2^]
(BH)_max_	maximum energy product [J/m^3^]
K	magnetocrystalline anisotropy energy [J/m^3^]
K_eff_	effective magnetic anisotropy constant [J/m^3^]
χ_p_	paramagnetic susceptibility [m^3^/kg]
K_d_	shape anisotropy constant [J/m^3^]
N_c_, N_a_	demagnetization factors
d_c_	critical diameter [m]
d_t_	threshold diameter [m]
l_K_	crystalline anisotropy length [m]
l_ex_	magnetostatic exchange length [m]
l_Keff_	effective anisotropy length [m]
A	exchange-stiffness constant [J/m]
T	temperature [K]
T_B_	blocking temperature [K]
T_C_	Curie temperature [K]
T_C_	Curie temperature [K]
T_0s_, T_0r_	characteristic temperatures above which magnetic ordering disappears [K]
