# Supplementary material for: Effect of Grinding and the Mill Type on Magnetic Properties of Carboxylated Multiwall Carbon Nanotubes
Source: Materials (Basel). 2021 Jul 20;14(14):4057. doi: 10.3390/ma14144057 (PMC8303930; doi:10.3390/ma14144057)
Supplement: Supplementary file 1 [file materials-14-04057-s001.zip › materials-1229393 _SI final_v2.pdf]

materials

Supplementary Materials

Effect of Grinding and the Mill Type on Magnetic Properties of Carboxylated Multiwall Carbon Nanotubes

Agnieszka Jamrozik [1], Janusz Przewoznik [2], Sonia Krysiak [2], Jozef Korecki [3], Grzegorz Trykowski [4], Artur Małolepszy [5], Leszek Stobiński [5] and Kvetoslava Burda [2,*]

1 Institute of Physical Chemistry, Polish Academy of Sciences, ul. Kasprzaka 44/52, 01-224 Warsaw, Poland; ajamrozik@ichf.edu.pl
2 Faculty of Physics and Applied Computer Science, AGH—University of Science and Technology; al. Mickiewicza 30, 30-059 Kraków, Poland; januszp@agh.edu.pl (J.P.); Sonia.Krysiak@fis.agh.edu.pl (S.K.)
3 Jerzy Haber Institute of Catalysis and Surface Chemistry, Polish Academy of Sciences, ul. Niezapominajek 8, 30-239 Krakow, Poland; korecki@agh.edu.pl
4 Faculty of Chemistry, Nicolaus Copernicus University in Torun, ul. Gagarina 7, 87-100 Torun, Poland; tryki@chem.umk.pl
5 Faculty of Chemical and Process Engineering, Warsaw University of Technology, ul. Waryńskiego 1, 00-645 Warsaw, Poland; Artur.Malolepszy@pw.edu.pl (A.M.); leszek.stobinski@pw.edu.pl (L.S.)
* Correspondence: kvetoslava.burda@fis.agh.edu.pl; Tel.: +48-126172991, Fax: +48-126340010

Citation: Jamrozik, A.;Przewoznik, J.; Krysiak, S.; Korecki, J.; Trykowski, G.; Małolepszy, A.; Stobiński, L.; Burda, K. Effect of Grinding and the Mill Type on Magnetic Properties of Carboxylated Multiwall Carbon Nanotubes. *Materials* **2021**, *14*, 4057. https://doi.org/10.3390/ma14144057

Academic Editor(s): Gueorgui Gueorguiev

Received: 5 May 2021
Accepted: 5 July 2021
Published: 20 July 2021

Publisher's Note: MDPI stays neutral with regard to jurisdictional claims in published maps and institutional affiliations.

Copyright: © 2021 by the authors. Licensee MDPI, Basel, Switzerland. This article is an open access article distributed under the terms and conditions of the Creative Commons Attribution (CC BY) license (http://creativecommons.org/licenses/by/4.0/).

Table 1. Hyperfine parameters fitted to the Mössbauer spectra measured at 85 K (*IS* – isomer shift related to the metallic Fe, *QS* – quadrupole splitting, H_{hf} – hyperfine magnetic field, ΔQ - quadrupole splitting distribution, ΔH – magnetic field distribution, *C* – relative contribution, Γ – line width).

Types of MWCNTs	**MWCNTs *as prepared***					**MWCNTs-COOH**				**MWCNTs-$COONH_4$**					
Component	**Fe_3C**	**α - Fe**	**Fe^{2+} in Fe_xO**	Fe oxides/ hydroxides/ ferrihydrates	Par **Fe^{2+}/Fe^{3+}**	**Fe_3C**	**α - Fe**	Fe oxides/ hydroxides/ ferrihydrates	Par **Fe^{2+}/Fe^{3+}**	**Fe_3C**	**α - Fe**	**Fe_xC_y**	**Fe^{2+} in Fe_xO**	Fe oxides/ hydroxides/ ferrihydrates	Par **Fe^{2+}/Fe^{3+}**
**control**															
C[%]	82.2±2.2	3.8±1.2	9.4±1.2	-	4.6±0.8	65.7±3.1	7.0±2/4	13.5±2.7	14.0±2.5	41.6±3.2	2.3±2.0	3.7±2.0	2.84±1.9	-	49.6±5
H_{hf} [T]	24.8<br>ΔH=0.6<br>p=0.8<br><br>24,5<br>ΔH=3.5<br>p=0.2<br><br><H>=24.7	33.6<br>ΔH=0.7	8.9<br>ΔH=1.1	-	-	25,1<br>ΔH=0.54<br>p=0.78<br><br>25.2<br>ΔH=3.0<br>p=0.22<br><br><H>=22.4	34.3<br>ΔH=1.1	8.5<br>ΔH=3.8	-	24.9<br>ΔH=0.46	33.8±1.5	13.6<br>ΔH=0.05	26.8<br>ΔH=0.1	-	-
ε /QS [mm/s]	< ε >=-0.005	0	0.02±0.04	-	0.73<br>ΔQ=0.37	< ε >=-0.009	0	-0.09±0.10	0.60<br>ΔQ=0.28	< ε >=-0.004	0	0.04±0.10	0.12±0.17	-	Q_1=0.69<br>ΔQ_1=0.22<br>(37.6%)<br><br>Q_2=1.20

															ΔQ_2=0.5<br>(12%)
IS [mm/s]	<IS>=0.20	-0.01±0.05	0.89±0.04	-	0.12±0.06	<IS>=0.21	0.06±0.05	0.39±0.11	0.40±0.03	<IS>=0.21	0.06±0.11	0.19±0.15	0.99±0.18	-	IS_1=0.38±0.02<br>IS_2=0.30±0.08
**agate mill**															
C[%]	82.1±2.1	6.1±1.0	3.4±1.1	8.6±0.6	-	82.6±5.8	6.7±3.0	-	10.6±1.9	48.5±2.1	3.1±1.6	-	4.8±1.8	9.0±2.3	34.5±2.5
H_{hf} [T]	25.1<br>ΔH=0.77<br>p=0.8<br><br>25.1<br>ΔH=6.8<br>p=0.2<br><br><H>=25.1	34,2<br>ΔH=0.78	17.2<br>ΔH=1.0	4.8<br>ΔH=1.6	-	25.1<br>ΔH=0.46<br>p=0.76<br><br>23.7<br>ΔH=3.3<br>p=0.24<br><br><H>=24.7	32.8<br>ΔH=0.03	-	-	24.9<br>ΔH=0.29	34.7<br>ΔH=0.76	-	23.7<br>ΔH=1.0	8.6<br>ΔH=1.7	-
ε /QS [mm/s]	< ε >=0.001	0	0.13±0.06	0.17±0.04	-	< ε >=0.001	0	-	0.57<br>ΔQS=0.25	< ε >=-0.011	0	-	0.23±0.11	0.09±0.10	0.71<br>ΔQS=0.30
IS [mm/s]	<IS>=0.20	-0.03±0.03	0.74±0.07	0.34±0.04	-	<IS>=0.21	0.12±0.10	-	0.27±0.08	<IS>=0.20	0.06±0.10	-	1.26±0.13	0.56±0.12	0.40±0.01
**steel mill**															
C[%]	83.9±2.2	6.4±1.0	3.3±0.7	-	6.4±0.7	15.6±1.4	12.8±1.7	42.3±3.5	29.2±2.8	44.3±2.7	5.8±1.9	-	5.2±1.6	12.1±2.1	32.6±3.7
H_{hf} [T]	24.8<br>ΔH=0.62<br>p=0.8	34.0<br>ΔH=0.73	9.4±0.4<br>ΔH=0.008	-	-	25.04<br>ΔH=0.67	32.2<br>ΔH=2.3	55.2<br>ΔH=3.2<br>(p=0.196)	-	24.8<br>ΔH=0.28	34.5<br>ΔH=0.5	-	24.1±0.7	8.3<br>ΔH=1.9	-

	23.2<br>ΔH=9.6<br>p=0.2<br><br><H>=24.5							45.0<br>ΔH=3.0<br>(p=0.047)<br><br>40.0<br>ΔH=3.0<br>(p=0.103)<br><br>35.0<br>ΔH=3.0<br>(p=0.041)<br><br>30.0<br>ΔH=3.0<br>(p=0.136)<br><br>20.0<br>ΔH=3.0<br>(p=0.155)<br><br>15.0<br>ΔH=3.0<br>(p=0.033)<br><br>10.0<br>ΔH=3.0							

								(p=0.289)<br><br><H>=29.0							
ε /QS [mm/s]	< ε >=-0.007	0	-0.03±0.05	-	0.81<br>ΔQS=0.28	0.006±0.010	0	0.03±0.04	QS_1=0.70<br>ΔQS_1=0.34<br>(22.3%)<br><br>QS_2=0.8<br>ΔQS_2=0.2<br>(7.1%)	< ε >=-0.019	0	-	0.18±0.11	0.18±0.15	0.71<br>ΔQS=0.25
IS [mm/s]	<IS>=0.19	-<br>0.002±0.03	0.94±0.06	-	0.30±0.02	0.20±0.01	-0.01±0.04	0.52±0.04	IS_1=0.34±0.03<br><br>IS_2=0.58±0.04	<IS>=0.21	0.08±0.10	-	0.97±0.11	0.66±0.12	0.40±0.02

Figure 1. Mössbauer spectra for (**a**) as prepared MWCNTs, (**b**) MWCNTs-COOH, and (**c**) MWCNTs-COONH4: left column – the control group, middle column – after using the agate mill, right column – after using the steel mill, measured at 220 K.

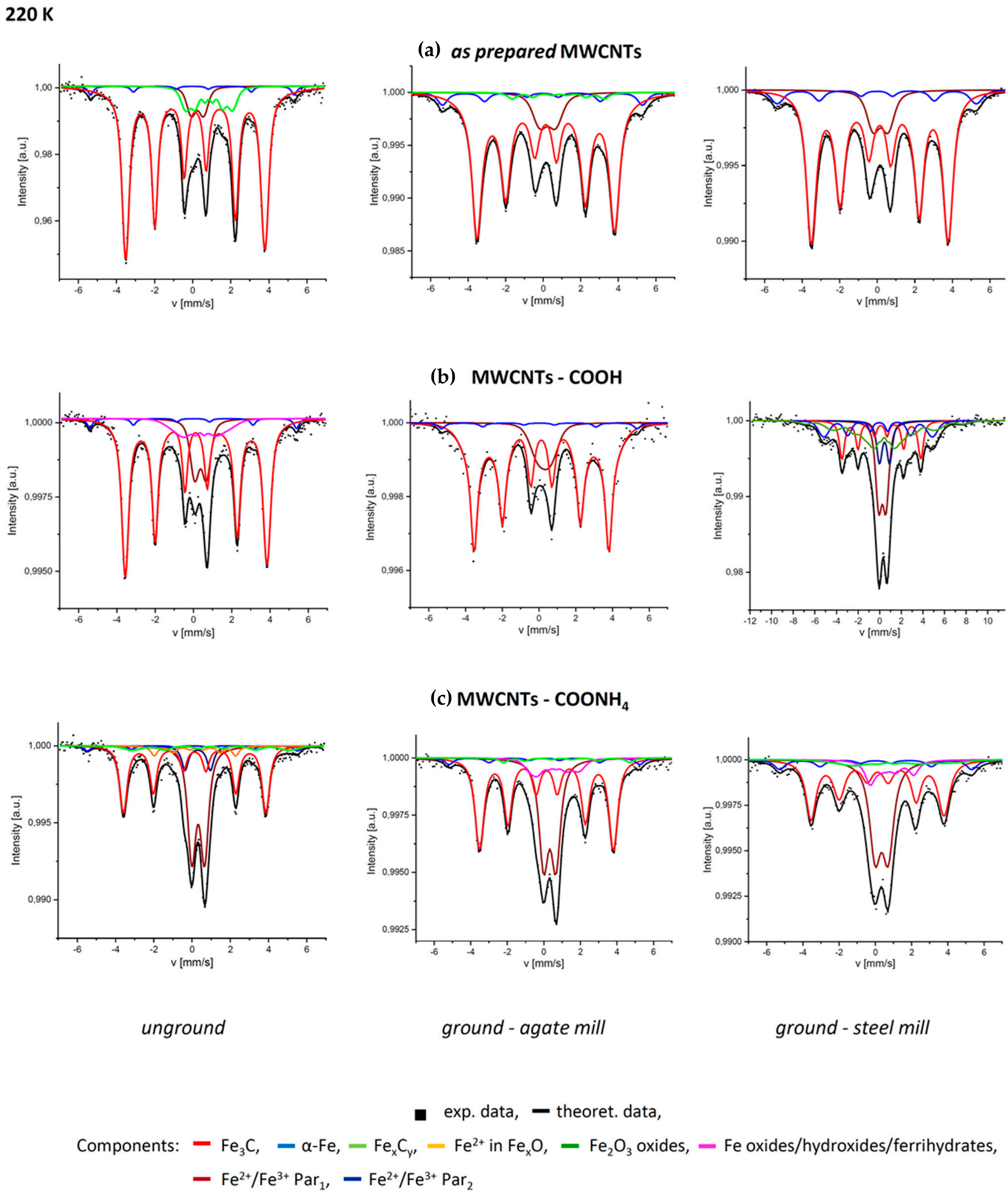

Table 2. Hyperfine parameters fitted to the Mössbauer spectra measured at 220 K (*IS* – isomer shift related to the metallic Fe, *QS* – quadrupole splitting, H_{hf} – hyperfine magnetic field, ΔQ - quadrupole splitting distribution, ΔH – magnetic field distribution, *C* – relative contribution, *Γ* – line width).

Types of MWCNTs	**MWCNTs *as prepared***				**MWCNTs-COOH**				**MWCNTs-COONH₄**					
Component	**Fe_3C**	**α - Fe**	**Fe^{2+} in Fe_xO**	Par **Fe^{2+}/Fe^{3+}**	**Fe_3C**	**α - Fe**	Fe oxides/ hydroxides/ ferrihydrates	Par **Fe^{2+}/Fe^{3+}**	**Fe_3C**	**α - Fe**	**Fe_xC_y**	**Fe^{2+} in Fe_xO**	Fe oxides/ hydroxides/ ferrihydrates	Par **Fe^{2+}/Fe^{3+}**
**control**														
C[%]	80.6±2.6	2.8±0.4	10.3±0.5	6.4±0.6	72.9±2.6	3.3±1.5	9.9±3.1	13.9±2.0	42.1±3.5	3.2±2.0	5.6±1.7	5.4±3.5	-	44.4±4.0
H_{hf} [T]	22.7<br>ΔH=0.66<br>p=0.77<br><br>19.5<br>ΔH=10.7<br>p=0.23	33.1<br>ΔH=0.07	7.6<br>ΔH=1.0	-	23.0<br>ΔH=0.60<br>p=0.82<br><br>19.9<br>ΔH=5.3<br>p=0.18	33.4<br>ΔH=0.007	7.7±2.0<br>ΔH=3.0	-	23.1<br>ΔH=0.39	Q=34.0<br>ΔQ=0.38	13.2±0.5	25.4<br>ΔQ=2.33	-	-

	<H>=22.0				<H>=22.4										
ε /QS [mm/s]	< ε >=0.002	0	0.01±0.01	Q=0.64<br>ΔQ=0.32	< ε >=-0.001	0	0.14±0.14	0.63<br>ΔQ=0.29	0.00±0.03	0	-0.05±0.14	0.007±0.175	-	Q_1=1.33±0.16<br>(7.8%)<br>Q_2=0.67<br>ΔQ_1=0.18<br>(36.6%)
IS [mm/s]	<IS>=0.13	-0.01±0.02	0.85±0.02	0.25±0.04	<IS>=0.13	-0.02±0.07	0.37±0.17	0.38±0.03	0.14±0.01	0.01±0.11	0.20±0.14	0.97±0.19	-	IS_1=0.30±0.04<br>IS_2=0.32±0.01
	**agate mill**														
C[%]	80.6±2.6	6.3±1.0	3.0±1.7	10.1±1.0	82.8±5.9	2.6±2.2	-	14.6±2.6	53.2±2.8	3.8±1.7	-	2.4±1.9	9.3±2.5	31.2±3.5
H_{hf} [T]	22.7	33.0	15.2	-	22.7	33.0	-	-	22.71	32.4	-	21.4	8.1±1.3	-

	ΔH=0.46<br>p=0.79<br>20.7<br>ΔH=7.1<br>p=0.21<br><H>=22..3	ΔH=0.5	ΔH=0.37		ΔH=0.001<br>p=0.57<br>22.7<br>ΔH=3.7<br>p=0.43<br><H>=22.7	ΔH=0.2			ΔH=0.55	ΔH=0.5		ΔH=0.007	ΔH=1.4	
ε /QS [mm/s]	< ε >=-0.004	0	-0.04±0.12	0.84<br>ΔQS=0.46	< ε >=0.002	0	-	0.71<br>ΔQS=0.66	< ε >=-0.007	0	-	0.14±0.28	0.07±0.12	0.67<br>ΔQS=0.21
IS [mm/s]	<IS>=0.13	-0.04±0.04	0.83±0.10	0.24±0.03	0.14±0.02	0.02±0.07	-	0.38±0.03	<IS>=0.15	0.03±0.10	-	1.1±0.3	0.53±0.14	0.33±0.02
**steel mill**														
C[%]	81.6±1.9	7.8±11	-	10.7±1.2	20.2±2.5	15.0±2.1	29.6±5.0	35.7±3.2	42.3±2.8	7.3±2.0	-	6.3±2.5	10.0±3.6	34.2±2.2
H_{hf} [T]	22.6±0.32<br>ΔH=0.59±0.11	32.9±0.4<br>ΔH=1.2±0.4	-	-	22.6<br>ΔH=0.66	31.1<br>ΔH=2.4	45<br>ΔH=3.0	-	22.8<br>ΔH=0.44	32.9<br>ΔH=0.5	-	17.6<br>ΔH=5.3	7.5<br>ΔH=0.01	-

	p=0.84<br><br>15.5±2.5<br>ΔH=6.2±2.4<br>p=0.16						(p=0.11)<br><br>35.0<br>ΔH=3.0<br>(p=0.039)<br><br>30.0<br>ΔH=3.0<br>(p=0.273)<br><br>20.0<br>ΔH=3.0<br>(p=0.076)<br><br>15.0<br>ΔH=3.0<br>(p=0.047)							

	<H>=21,5						10.0<br>ΔH=3.0<br>(p=0.455)<br><H>=21.3							
ε /QS [mm/s]	< ε >=-0.03	0	-	Q=0.79<br>ΔQ=0.38	-0.02±0.02	0	0.0	QS_1=0.67<br>ΔQS_1=0.3<br>(24.5%)<br>QS_2=0.94<br>ΔQS_2=0.24<br>(11.2%)	< ε >=-0.003	0	-	0.25 ± 0.33	0.23 ± 0.19	0.72<br>ΔQS = 0.2
IS [mm/s]	<IS> = 0.15	-0.03±0.05	-	0.18±0.04	0.13±0.03	-0.10±0.11	0.41±0.13	IS_1=0.25±0.4<br>IS_2=0.45±0.22	<IS>=0.13	0.02±0.1	-	0.87±0.15	0.68±0.15	0.35±0.02

Figure 2. Mössbauer spectra for (**a**) as prepared MWCNTs, (**b**) MWCNTs-COOH, and (**c**) MWCNTs-COONH4: left column – the control group, middle column – after using the agate mill, right column – after using the steel mill, measured at 295 K.

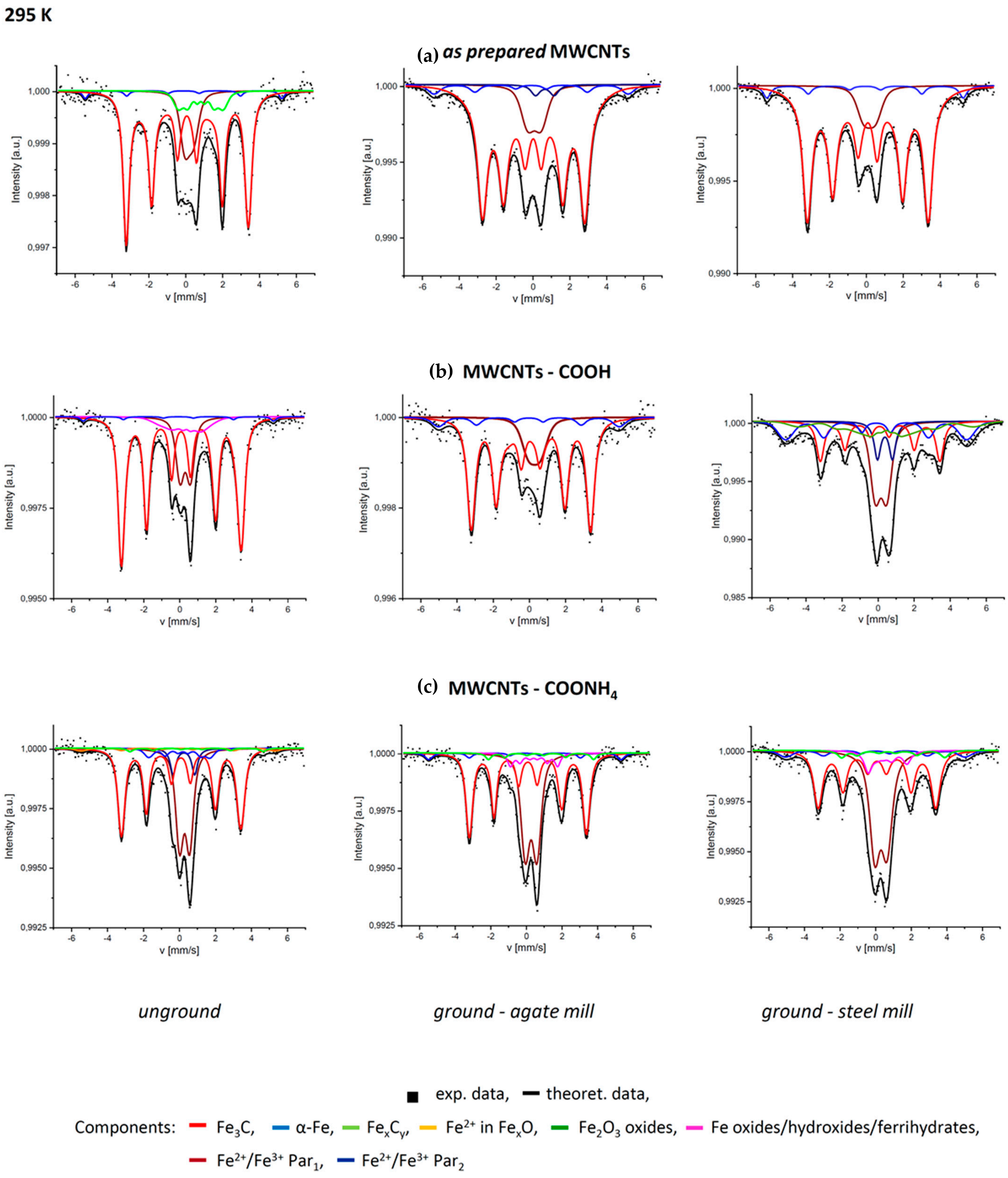

Table 3. Hyperfine parameters fitted to the Mössbauer spectra measured at 295 K (*IS* – isomer shift related to the metallic Fe, *QS* – quadrupole splitting, *H*hf – hyperfine magnetic field, *ΔQ* - quadrupole splitting distribution, *ΔH* – magnetic field distribution, *C* – relative contribution, *Γ* – line width).

Component	**Fe_3C**	**α - Fe**	**Fe^{2+} in Fe_xO**	Par **Fe^{2+}/Fe^{3+}**	**Fe_3C**	**α - Fe**	Fe oxides/ hydroxides/ ferrihydrates	Par **Fe^{2+}/Fe^{3+}**	**Fe_3C**	**α - Fe**	**Fe_xC_y**	**Fe^{2+} in Fe_xO**	Fe oxides/ hydroxides/ ferrihydrates	Par **Fe^{2+}/Fe^{3+}**
**control**														
C[%]	73.5±3.5	2.9±0.8	9.4±1.3	14.2±3.5	72.5±3.1	1.8±1.0	9.1±3.0	16.8±2.6	52.3±3.3	2.4±2.0	6.3±2.5	1.8±1.5	-	37.4±3.7
H_{hf} [T]	20.5<br>ΔH=0.41<br>p=0.78<br><br>13.7<br>ΔH=5.0<br>p=0.22<br><br><H>=19.0	33.0<br>ΔH=0.03	H=7.5<br>ΔH=1.05	-	20.7<br>ΔH=0.49<br>p=0.77<br><br>19.6<br>ΔH=1.08<br>p=0.23<br><br><H>=20.4	32.7<br>ΔH=0.002	7.4<br>ΔH=3.0	-	20.6<br>ΔH=0.61	H=33.4<br>ΔH=1.3	H=10.5<br>ΔH=0.9	22.3<br>ΔH=0.05	-	-
ε /QS [mm/s]	< ε >=-0.029	0	0.00±0.12	Q=0.48<br>ΔQ=0.36	< ε >=0.001		-0.07±0.17	0.57<br>ΔQ=0.26	< ε >=0.006	0	-0.10±0.10	0.13±0.26	-	QS_1= 0.56<br>$ΔQS_1$=0.22<br>(30.5%)<br><br>QS_2=1.2<br>$ΔQS_2$=0.004<br>(6.9%)
IS [mm/s]	<IS>=0.04	-0.12±0.22	0.82±0.16	<IS>=0.18	<IS>=0.07	-0.05±0.20	0.31±0.15	0.30±0.03	<IS> =0.09	-0.10±0.11		0.82±0.26	-	IS_1=0.29±0.01

											0.09±0.13			IS$_2$=0.25±0.05
**agate mill**														
C[%]	80.4±1.5	5.3±1.3	-	14.3±3.0	76.3±1.1	8.0±3.5	-	15.7±2.9	49.7±3.0	4.2±1.5	-	3.4±.1.6	6.8±2.1	35.9±2.0
H_{hf} [T]	17.23<br>ΔH=0.53<br>p=0.63<br><br>15.1<br>ΔH=6.1<br>p=0.37<br><br><H>=16.4	32.7±0.6<br>ΔH=1.2	-	-	20.51<br>ΔH=0.41<br>p=0.90<br><br>10.5<br>ΔH=1.2<br>p=0.10<br><br><H>=19.4	31.0<br>ΔH=1.4	-	-	20.4<br>ΔH=0.50	33.4<br>ΔH=0.6	-	18.3<br>ΔH=0.04	8.2<br>ΔH=0.02	-
ε /QS [mm/s]	< ε >=0.02	0	-	QS$_1$= 0.73<br>ΔQS$_1$=0.45<br>(11.9%)<br><br>QS$_2$=1.0<br>ΔQS$_2$=0.16<br>(2.4%)	< ε >=0.03	0	-	0.65<br>ΔQS=0.52	< ε >=-0.001	0	-	0.16±0.14	0.01±0.07	0.65<br>ΔQS=0.29
IS [mm/s]	<IS>=0.03	-0.10±0.09	-	IS$_1$=0.08±0.04<br><br>IS$_2$=0.64±0.12	<IS>=0.07	-0.03±0.17	-	0.28±0.08	<IS>=0.09	-0.08±0.10	-	0.66±0.14	0.42±0.08	0.27±0.01
**steel mill**														
C[%]	83.8±2.3	5.6±1.5	-	10.6±2.4	24.3±2.0	19.4±1.9	18.6±3.5	37.6±3.2	40.9±3.8	6.5±2.0	-	4.8±.2.0	8.6±1.6	39.2±3.8

H_{hf} [T]	20.35<br>ΔH=0.51<br>p=0.71<br><br>17.1<br>ΔH=6.7<br>p=0.29<br><br><H>=19.4	33.0<br>ΔH=0.03	-	-	20.6<br>ΔH=0.44	31.1<br>ΔH=2.0	30.0<br>ΔH=3.0<br>(p=0.40)<br><br>15.0<br>ΔH=3.0<br>(p=0.08)<br><br>10.0<br>ΔH=3.0<br>(p=0.52)<br><br><H>=18.3	-	20.5±0.1<br>ΔH=0.48	31.6±1.5<br>ΔH=1.97	-	18.0<br>ΔH=0.02	6.8<br>ΔH=0.05	-
ε /QS<br>[mm/s]	< ε >=0.000	0	-	Q=0.63<br>ΔQ=0.48	0.01±0.01	0	0.0	QS_1=0.63<br>ΔQS_1=0.33<br>(27.3%)<br><br>QS_2=0.83<br>ΔQS_2=0.02<br>(10.3%)	-0.001±0.025	0	-	0.17±0.16	0.20±0.18	0.68<br>ΔQS=0.28
IS [mm/s]	<IS> = 0.08	-0.07±0.07	-	0.18±0.05	0.10±0.01	-0.10±0.11	0.44±0.12	IS_1=0.16±0.02<br><br>IS_2=0.38±0.03	0.07±0.03	-0.1±0.1	-	0.82±0.16	0.45±0.10	0.26±0.05

Table 4. Metal and semimetal concentrations of MWCNTs obtained by use of ICP-OES method. Concentrations are given in [μg/g]. n.d. – below the detection limit.

Element	*as prepared* MWCNTs	MWCNTs-COOH	MWCNTs-COONH$_4$
		control	
Al	154 ± 2	35.5 ± 0.1	11.9 ± 0.1
As	69.9 ± 1.4	11.7 ± 0.5	14.4 ± 0.8
B	126 ± 4	19.8 ± 0.1	24 ± 0.2
Ca	30.8 ± 0.8	143 ± 2	41 ± 1
Cd	2.1 ± 0.4	n.d.	n.d.
Cr	4.0 ± 0.7	n.d.	n.d.
Cu	6.2 ± 0.3	0.53 ± 0.01	1.1 ± 0.1
Fe	342 ± 2	54.4 ± 0.2	56.9 ± 0.3
Hg	250 ± 38	4.2 ± 1.1	4.9 ± 3.0
K	5.8 ± 1.5	12.4 ± 0.8	3.1 ± 0.3
Mg	n.d.	4.26 ± 0.05	1.64 ± 0.06
Mo	3.2 ± 0.5	n.d.	n.d.
Na	21.4 ± 0.5	12.1 ± 0.3	5.6 ± 0.1
Sb	29 ± 3	1.4 ± 0.5	n.d.
Si	23.9 ± 0.5	19.2 ± 0.9	12.5 ± 1.1
V	1.6 ± 0.2	n.d.	n.d.
W	15.2 ± 1.7	n.d.	1.3 ± 0.1
		agate mill*	
Fe	330 ± 3	53.1 ± 0.4	54.6 ± 0.4
		steel mill*	
Fe	334 ± 4	54.0 ± 0.4	55.5 ± 0.4

*The contents of other elements in the ground samples did not change.

Figure 3. Temperature dependencies of the magnetic moment (μ) measured in the field of 4 T for MWCNTs-COOH obtained from MWCNTs prepared in the agate (blue squares) and steel (red circles) mill. Empty symbols denote the data as measured and full symbols correspond to the values corrected for the carbon contribution.

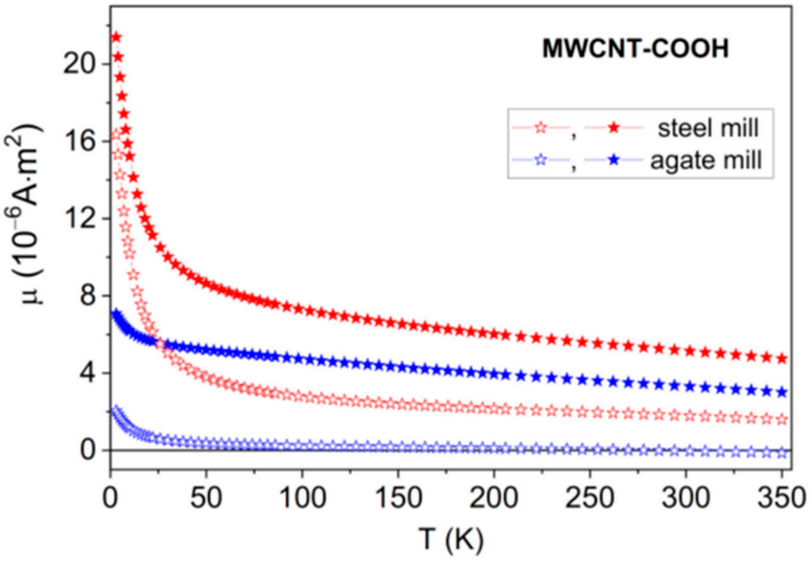

Figure 4. TEM image of a large nanoparticle in MWCNTs-COOH and an experimental evidence (EDX measurements) that it contains Fe, C and O is given.

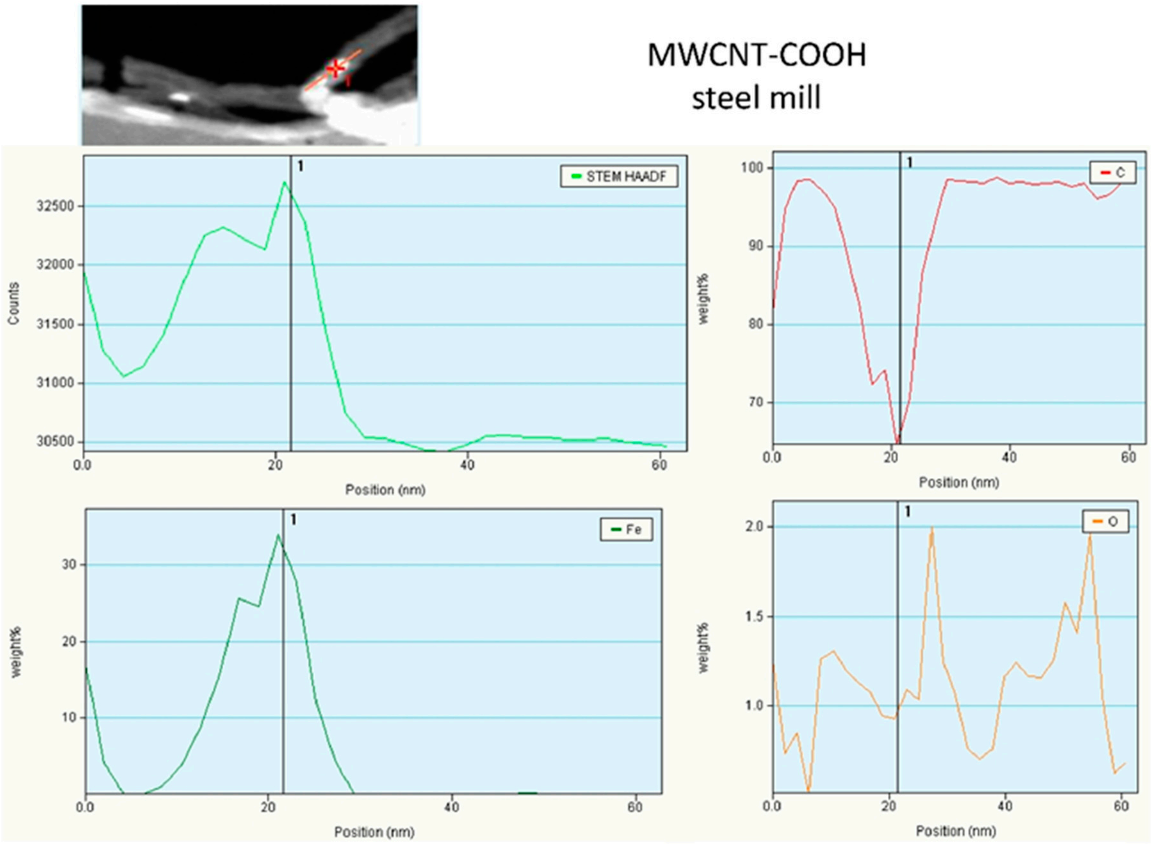
MWCNT-COOH
steel mill
STEM HAADF
Counts
Position (nm)
C
weight%
Fe
O
